# *De novo* assembly of a transcriptome from the eggs and early embryos of *Astropecten aranciacus*

**DOI:** 10.1371/journal.pone.0184090

**Published:** 2017-09-05

**Authors:** Francesco Musacchia, Filip Vasilev, Marco Borra, Elio Biffali, Remo Sanges, Luigia Santella, Jong Tai Chun

**Affiliations:** 1 Department of Biology and Evolution of Marine Organisms, *Stazione Zoologica Anton Dohrn*, Napoli, Italy; 2 The Molecular Biology and Bioinformatics Unit, *Stazione Zoologica Anton Dohrn*, Napoli, Italy; Chang Gung University, TAIWAN

## Abstract

Starfish have been instrumental in many fields of biological and ecological research. Oocytes of *Astropecten aranciacus*, a common species native to the Mediterranean Sea and the East Atlantic, have long been used as an experimental model to study meiotic maturation, fertilization, intracellular Ca^2+^ signaling, and cell cycle controls. However, investigation of the underlying molecular mechanisms has often been hampered by the overall lack of DNA or protein sequences for the species. In this study, we have assembled a transcriptome for this species from the oocytes, eggs, zygotes, and early embryos, which are known to have the highest RNA sequence complexity. Annotation of the transcriptome identified over 32,000 transcripts including the ones that encode 13 distinct cyclins and as many cyclin-dependent kinases (CDK), as well as the expected components of intracellular Ca^2+^ signaling toolkit. Although the mRNAs of cyclin and CDK families did not undergo significant abundance changes through the stages from oocyte to early embryo, as judged by real-time PCR, the transcript encoding Mos, a negative regulator of mitotic cell cycle, was drastically reduced during the period of rapid cleavages. Molecular phylogenetic analysis using the homologous amino acid sequences of cytochrome oxidase subunit I from *A*. *aranciacus* and 30 other starfish species indicated that Paxillosida, to which *A*. *aranciacus* belongs, is not likely to be the most basal order in Asteroidea. Taken together, the first transcriptome we assembled in this species is expected to enable us to perform comparative studies and to design gene-specific molecular tools with which to tackle long-standing biological questions.

## Introduction

Starfish (class Asteroidea) represent one of the most successful life forms in the animal kingdom. After their ancestors emerged circa 500 million years ago, as many as 1,890 diverse species of starfish are thriving today on the seabed all over the world [1–3]. As general predators living on bivalves, microalgae and corals, starfish have profound impacts on their ecological communities, and often inflict serious harms on commercially valuable marine resources such as mussels, clams, and crabs [4–6].

In addition to their significant role as a ‘keystone species’ in ecology, starfish have also served as an important animal model in various fields of biological research. Starfish have long been noted for their remarkable capability of regenerating their body parts [7], and their embryonic development has been well described [8]. As starfish belong to Echinodermata, which is a phylum of deuterostome along with Chordata, findings in starfish would provide insights into biology of vertebrate animals.

Starfish have been particularly useful for cell biology, as their gonads provide a population of synchronized cells that are plenty and easy to access. Starfish oocytes are electrically excitable and highly apt for intracellular recording owing to their large size (200–350 μm in diameter) and resilient cell surface [9–11]. Optically transparent, these cells are also suitable for microinjection of fluorescent dyes that visualize changes of organelles, cytoskeleton, chromosomes, and free calcium ion levels during meiotic maturation, egg activation, and cytokinesis [12–22]. Thus, starfish oocytes have served as an experimental model system highly useful in addressing fundamental questions in cell biology on a single cell level.

For example, starfish oocytes provide an invaluable opportunity to study meiotic cell cycle controls. In breeding seasons, ovaries of female starfish are full of oocytes arrested at the first prophase of meiosis, marked by the presence of the tetraploid (4n) nucleus termed ‘germinal vesicle (GV).’ At spawning, the oocytes are unblocked from the cell cycle arrest to resume meiotic progression in response to the maturation hormone. With 1-methyladenine (1-MA) being identified as the oocyte maturation hormone virtually in all species of starfish [23], meiotic resumption can be easily induced by adding the hormone to the oocytes in seawater. As the oocytes reenter the cell cycle in response to 1-MA, a series of cytological changes take place, which include accelerated reorganization of the actin cytoskeleton, prompt increase of the intracellular Ca^2+^ level, changes of the phosphorylation state of proteins, increase of protein synthesis, relocation of the cortical granules, and shift of the membrane potential [24–30]. The hallmark of meiotic maturation is the germinal vesicle breakdown (GVBD), which represents dissolution of the nuclear envelope, and the consequent intermixing of nucleoplasm and cytoplasm is essential for a normal Ca^2+^ response at fertilization [31]. The major down-stream effector of the hormone that plays the central role in orchestrating the G2-metaphase transition is the maturation-promoting factor (MPF), which was identified in starfish oocytes as a heterodimer of CDK1 (cdc2) and cyclin B [32]. In the somatic and germ cells of starfish, however, not much has been known about the molecular repertoire of cyclin and CDK families and their combinatorial diversity.

Meiotic progression and the following intracellular changes in the eggs are finely coordinated with fertilization events, but there are subtle differences depending on the species. Whereas sea urchin eggs are spawned and fertilized after the completion of meiosis, the natural timing of fertilization for starfish is known to be between GVBD and the extrusion of the first polar body [33,34]. This disparity is quite remarkable when considering that both starfish and sea urchin belong to the same phylum and that fertilization is such a crucial event for survival of the species. As aforementioned, meiosis in the oocyte involves a series of cytological changes, yet fertilization triggers another series of comparable but distinct physicochemical changes in the eggs that are collectively called ‘egg activation’ [35–37]. The changes following gametes fusion may also vary considerably from species to species. Virtually in all animal species, however, fertilization is accompanied by Ca^2+^ fluxes and waves in the egg. However, it bears emphasis that the precise mechanisms by which the Ca^2+^ signal is produced and utilized are not completely understood [38–42].

Although the generation of intracellular Ca^2+^ waves in fertilized eggs is a universal phenomenon, the molecular components of the Ca^2+^ signaling machinery may vary markedly depending on the animal species. An instant example is found in phospholipase C (PLC), the enzyme that synthesizes inositol 1,4,5-trisphosphate (InsP_3_), the major intracellular Ca^2+^-releasing second messenger. PLC exists as multiple isozymes such as ß, ɣ, δ, and ε, which transduce different signaling cues owing to the unique combination of various functional domains [43]. In mammals, a sperm-specific isozyme PLC-ζ was discovered and suggested as the trigger of Ca^2+^ waves in fertilized eggs [44]. However, PLC-ζ does not exist in the genome of sea urchin (*Strongylocentrotus purpuratus*), suggesting that the triggering mechanism of the Ca^2+^ waves in the fertilized eggs of echinoderm has to be quite different from that of the mammalian eggs [45]. Besides InsP_3_, generation and propagation of the intracellular Ca^2+^ waves in echinoderm eggs at fertilization have been recapitulated with the concerted action of additional Ca^2+^-mobilizing second messengers such as cyclic-ADP ribose (cADPr) and nicotinic acid adenine dinucleotide phosphate (NAADP) [31,46–48]. Interestingly, eggs from different species of starfish respond to these Ca^2+^-mobilizing second messengers with selective sensitivity. Whereas NAADP evokes an intense Ca^2+^ response in the eggs of *Asterina pectinifera* (also called *Patiria pectinifera*), it produces only a modest Ca^2+^ increase in the eggs of *A*. *aranciacus*. Conversely, in the case of cADPr, the opposite is true for the two species of starfish [16,49,50]. In addition, the abrupt increases of Ca^2+^ in oocytes at the onset of meiotic resumption and during GVBD are also remarkably different in the two species of starfish [22]. Furthermore, it has been demonstrated that eggs of *A*. *aranciacus* release Ca^2+^ from internal stores in response to an actin drug latrunculin A, which binds to actin monomers and inhibits their polymerization to change the cytoskeletal structure. This intriguing intracellular Ca^2+^ increase that resembles the Ca^2+^ signals in fertilized eggs and recurs for hours has been observed neither in the GV-stage oocytes nor in the eggs of other species of starfish [51]. These evident differences in the pattern or mechanism of intracellular Ca^2+^ signaling in the eggs of different animal species may arise from species-specific differences in the molecular makeup of the Ca^2+^ signaling toolkits or in their layout inside the cells [52–54].

Despite the essential attributes apt for studying various aspects of biology, scientific research using starfish oocytes has often been hampered largely because of the lack of comprehensive DNA and protein sequence information. This is particularly true for *A*. *aranciacus*, the Mediterranean species of starfish that we and other laboratories in Europe have used as an animal model for many decades. In the NCBI database, for example, merely 158 nucleic acid sequences have been deposited for this species to date. In contrast, two other starfish species being utilized as model organisms for developmental biology have accumulated a substantial amount of sequence information for DNA and protein. *A*. *pectinifera*, a Northern Pacific species easily found in Japan, China, Russia, and Korea, has over 118,000 nucleic acid sequences in the NCBI database amassed from cDNA libraries of unfertilized eggs and the embryos at gastrula stage. On the other hand, the genome of *Patiria miniata*, a species living on the other side of the Pacific near Alaska and California, has been sequenced and assembled (https://www.ncbi.nlm.nih.gov/genome/?term=Patiria+miniata). The results of ongoing annotation for *P*. *miniata* genome and the genetic sequence information of some other echinoderm species are available at the echinoderm genomic database EchinoBase (www.echinobase.org). Likewise, genomes of two starfish species living in the Indo-Pacific near Australia and New Zealand have been recently sequenced and assembled: *Acanthaster planci* (https://www.ncbi.nlm.nih.gov/assembly/GCA_001949145.1) and *Patiriella regularis* (https://www.ncbi.nlm.nih.gov/assembly/GCA_900067625.1). Hence, compared with those biologically and ecologically important species in other regions of the world, the genome or transcriptome of the starfish species being used as biological model organisms in European seas and ocean has been far less explored.

In an attempt to crack this barrier, we have performed deep sequencing of RNA from the GV-stage oocytes, mature eggs, zygotes and early embryos of *A*. *aranciacus* in this study. Oocytes contain stockpiles of mRNAs and ribosomes required for early embryonic development, and have been known as the cell type manifesting the highest RNA sequence complexity [55,56]. The species we chose, *A*. *aranciacus*, belongs to the order Paxillosida in the taxonomy of Asteroidea. This group of starfish was considered a primitive order as judged by their inability to evert the cardiac stomach and the lack of the brachiolaria stage in their larval development. However, their fossil records are recovered later in the geological time table in comparison with the other starfish species, while classifications based on DNA sequences of a few genes have placed them as an order either primitive or derived in the phylogeny [57–59]. Hence, acquisition of comprehensive genetic information would not only facilitate experimental approaches to the biology of starfish oocytes, but also assist in resolving the controversial issues regarding ordinal classification of starfish.

## Materials and methods

### Animals, RNA preparation, and RNA-seq

During the breeding season, starfish (*A*. *aranciacus*) were captured in the sea near Gaeta, Italy, under the authorization of the *Ministero delle Infrastrutture e dei Trasporti Capitaneria di Gaeta*. The animals were not endangered or protected species, and captured in locations that were not privately-owned or protected in any way. Animals were kept at 16°C, and all experimental procedures were in compliance with the guidelines of the European Union Directive 86/609. Oocytes at the germinal vesicle (GV) stage were isolated as was previously described [21]. To obtain mature eggs, the oocytes were exposed to 1-MA (10 μM) for 70 min in filtered seawater. The eggs were then fertilized with spermatozoa from one male animal and allowed to undergo cleavage for 4 h and 20 min. Functional integrity of the cells was monitored by light microscopy, and most fertilized eggs appeared viable and reached the 8-cell stage on the average. At each stage, i.e. GV-stage oocytes, mature eggs, zygotes (20 min post-fertilization), and early embryos (4 h and 20 min post-fertilization), aliquots of cells were taken and rinsed in filtered seawater for RNA extraction. RNA was prepared from each sample by use the RNAquous-Micro kit (Ambion) and treated with DNAse following the manufacturer’s instruction. The RNA samples were quantified by spectrophotometry (NanoDrop ND1000, Thermo Scientific), and its quality was assessed on RNA LabChip (Aligent bioanalyzer 2100, Aligent Technologies). The RNA samples derived from eggs of four females fertilized with sperm of the same male displayed high quality, as their λ_260nm_/λ_280nm_ absorption ratio did not deviate from the range of 1.95–2.05, and their RIN (RNA Integrity Number) from the range of 8.8–10 in all cases. For RNA-seq, total RNA (> 6 μg for each sample) were sent to EMBL-GeneCore (Heidelberg, Germany) for the procedures of cDNA synthesis, fragmentation, library construction, and massive parallel sequencing by use of the Illumina system. RNA-seq for biological quadruple samples were all processed separately except for the GV stage oocytes, which were pooled together.

### Data filtering and *de novo* assembly of the transcriptome

Raw sequencing data were subjected to adapter trimming and quality filtering by use of the preprocessing tool Trimmomatic [60] with the following parameters: ILLUMINACLIP:illumina_adapters.fa:2:40:15 LEADING:3 TRAILING:3 SLIDINGWINDOW:3:20 MINLEN:25. More than 80% of the reads were retained on average. The filtered reads were normalized using the script normalize_by_kmer_coverage.pl (parameters:—seqType fq—JM 240G —max_cov 30), and assembled with the Trinity software version r2013_08_14 (parameters:—seqType fq—JM 240G —inchworm_cpu 24—bflyHeapSpaceInit 24G —bflyHeapSpaceMax 240G —bflyCalculateCPU—CPU 24—jaccard_clip—min_kmer_cov 2) [61]. The assembled contigs were further clustered by use of the software CD-HIT-EST [62]. To obtain quantitative information, we mapped the raw reads to the filtered assembled transcripts using Bowtie (parameters: -t -q -p 24—chunkmbs 10240—maxins 500 –S). To remove sequences not having enough representative reads, only those transcripts with the ‘count per million (cpm)’ values greater than 1 were retained for further analyses. After this series of filterings, the complete transcriptome contained 64,388 assembled sequences, which we will refer to as ‘transcripts’ for the sake of discussion. The completeness of the transcriptome was assessed by use of the software CEGMA [63].

### Functional annotation and differential expression analyses

To annotate the transcripts, we used the Annocript pipeline [64] which runs different BLAST programs to align sequences against the UniProt database: *i*) BLASTP against the UniRef90 and SwissProt databases (parameters: word_size = 4 evalue = 10^−5^ num_descriptions = 5 num_alignments = 5 threshold = 18), *ii*) RPSTBLASTN to align the transcripts against the Conserved Domains Database (CDD) (parameters: evalue = 10^−5^, num_descriptions = 20, num_alignments = 20). The results produced comprehensive lists of multiple sequences annotated on the basis of alignment models for ancient domains and full-length proteins derived from Pfam, SMART, COG, PRK, and TIGRFAM [65]. In addition, we used TBLASTN to obtain alignments against other non-coding RNAs (rRNA, tRNA, snRNA, snoRNA, miRNA) exploiting the database Rfam integrated with a customized set of NCBI ribosomal RNAs (parameters: evalue = 10^−5^, num_descriptions = 1, num_alignments = 1. To characterize the mRNA population, transcripts were further annotated using the Gene Ontology (GO) database [66], the Enzyme Commission IDs [67] and UniPathways [68]. The R package edgeR [69] was used to perform differential expression analysis of the genes for each pair of sequential stages of development, i.e. GV-stage oocytes versus mature egg, mature eggs versus zygotes, and zygotes versus early embryos. Transcripts were considered differentially expressed if the ‘False Discovery Rate (FDR)’ was less or equal to 0.05 and the fold change greater than two. When comparing expression levels of different transcripts in the sample, we also utilized RPKM (reads per kilobase of transcripts per million mapped reads) to compensate for the differences in transcript length, by use of the rpkm() function included in the edgeR package.

### Validation of transcripts by RT-PCR and DNA sequencing

RNA was extracted from GV-stage oocytes, mature eggs, zygotes, and early embryos derived from eggs of four females fertilized with sperm of the same male, and converted to cDNA by use of SuperScript VILO MasterMix (Invitrogen) with random hexamers following the manufacturer’s instruction. The RNA samples showed no sign of contamination from genomic DNA, as judged by the total elimination of RT-PCR signals by RNAse treatment prior to cDNA synthesis [70] (S1 Fig). Stocks of cDNA obtained from 2.5 μg RNA in 20 μl were diluted 40-fold and used for PCR to evaluate the primers elected from 32 different transcripts in the transcriptome (S1 Table). Initially, the target regions of the transcripts were amplified by a PCR thermal cycler EP S (Eppendorf) in 96-well plates, following the standard procedure for Taq DNA polymerase: 30 cycles at 95°C for 1 min, 52°C for 1 min, and 72°C for 1 min. The resulting amplicons were resolved by electrophoresis (2% agarose gel) and purified by use of QIAquick gel extraction kit (QIAGEN). The eluted DNA (15 nM) was subjected to direct DNA sequencing (BigDye Terminator Cycle Sequencing technology, Applied Biosystems) using the same primers (4.5 μM) for PCR. For real-time qPCR, reaction mixtures were prepared in 384-well plates by use of Beckman Coulter’s Biomek FX Laboratory Automation Workstation equipped with the ORCA robotic arm (Beckman Coulter, Fullerton, CA). Each reaction well contained diluted cDNA, a pair of primers (0.25 μM, each), and Fast SYBR Green Master Mix with ROX (Applied Biosystems). Reactions were run in a ViiA 7 Real-Time PCR System (Applied Biosystems). PCR for each developmental stage was performed in biological quadruplicate, and each of them in technical triplicate. The reaction cycle was repeated 40 times as follows: *i*) denaturation at 95°C for 20 s, *ii*) annealing and elongation at 60°C for 1 min. At the end of the PCR cycles, melting-curve analysis was performed between 95°C and 60°C using the machine-installed program to assess specificity of the primer pairs. The PCR data were analyzed using the ViiA 7 Software and Microsoft Excel 2010. Reference genes were selected by empirical data. Merging the transcripts annotation table and their raw counts, only transcripts with more than 5X coverage in all replicates were considered. Counts per million (CPM) were computed for all transcripts, and coefficient of variation (CV) was calculated from the formula RS/RM, where RS is the standard deviation and RM the mean of the CPM values. The transcripts annotated in SwissProt or Conserved Domain Databases with highest Query- and Hit-Coverage while scoring lowest CV were elected as reference genes: Histone deacetylase 1, Actin-related protein 2/3 complex subunit 5 (ARP 2/3), Protein SEC13 homolog (SEC 13), U5 small nuclear RNP helicase (snRNP helicase), Mediator of RNA polymerase II subunit 12-like (RNA pol II modulator), Pre-mRNA splicing factor 8 (splicing factor 5), and Elongation factor 1-alpha (EF1A). In the given experimental conditions of qPCR, the thresholds cycles (CT) for the reference genes were arrived as follows: snRNP helicase, CT = about 20; SEC 13, 21; RNA pol II modulator, 23; ARP 2/3, 24; EEA1, 18; Splicing factor 5, 19. Except for Histone deacetylase 1, each reference gene produced comparable and fair results with tightly uniform expression levels throughout the developmental stages. To minimize operational errors in overcrowded experiments, we used Splicing factor 5 as the representative reference gene, as its CT was close to the median of the CT values of all target genes that ranged from 16 to 24. Thus, the relative abundance of each target transcript in a given RNA sample was estimated by comparing the values of the threshold cycles (CT) for the target genes with that of the reference gene (splicing factor 5), which showed nearly constant CT values in all samples. The fold changes of a given transcript during the developmental transition were calculated using the 2^-ΔΔC^_T_ method [71].

### Molecular phylogenetic analysis

The transcript of the mitochondrial gene encoding cytochrome c oxidase subunit I in *A*. *aranciacus* (comp182578) was analyzed with Virtual Ribosome [72] by use of the mitochondrial codons of echinoderm. After determining the coding region, homologous DNA sequences in other species of starfish were obtained from GenBank. The region commonly available in the majority of the species was defined (see Results), and the deduced amino acid sequences from various species were subjected to multiple alignment by use of MAFFT version 7 (http://mafft.cbrc.jp/alignment/server/) with default parameters. The alignment data were converted into a phylogenetic tree by the neighbor-joining algorithm [73] with the bootstrap resampling number set at 100. The resulting tree was visualized with Phylo.io 1.0 [74].

### Statistical analysis

The average and variation of the data were reported as ‘mean ± standard deviation (SD)’ in all cases in this manuscript. Oneway ANOVA and t-tests were performed by use of Prism 3.0 (GraphPad Software), and P<0.05 was considered as statistically significant. For ANOVA results showing P<0.05, statistical significance of the difference between the comprising groups was assessed by *post hoc* tests.

## Results

### Acquisition of the reference transcriptome

Sequencing of the cDNA from the GV-stage oocytes, eggs, zygotes, and early embryos of *A*. *aranciacus* led to collection of 4.8 x 10^8^ paired-end reads made of 104 bp. After filtering with Trimmomatic [60], 372,263,524 reads were assembled using the software Trinity, which led to 68,218 contigs with a N50 value of 1,755 and the average contig length of 1,368 bp. The transcriptome was clustered by use of CD-Hit and filtered further on the basis of the criterion that the transcripts should be expressed in at least 2 samples displaying more than 1 count per million (cpm). Thus, we finally obtained 64,388 transcripts after assembly and filtering, which were defined as our ‘reference transcriptome’ of *A*. *aranciacus*. Then, completeness analysis of the assembly was performed exploiting CEGMA (Core Eukaryotic Gene-Mapping Approach, v2.5), which estimates the presence of the “core” genes using a representative set of 458 Core Eukaryotic Genes (CEGs) that are highly conserved among eukaryotic species. The results of CEGMA showed that 94.4% of the core genes were present within the *A*. *aranciacus* transcripts over 70% of the protein length (complete alignment), a clear indication of fairly good completeness of the transcriptome. Fastq files of the assembled reads for each sample are now available in ArrayExpress database under the accession number E-MTAB-5679 (https://www.ebi.ac.uk/arrayexpress/experiments/E-MTAB-5679).

### Comparison of the *A*. *aranciacus* reference transcriptome with the existing genetic sequence databases

The transcriptome was annotated with Annocript software that performs alignments against the UniProt databases (see Methods and materials). In this way, we have annotated 32,783 transcripts that exhibited at least one hit in the databases of SwissProt, UniRef proteins, Conserved Domains Database (CDD), or Rfam. The minimum and maximum lengths of transcripts were 201 and 34,228 nucleotides, respectively, and the mean of their CG contents was 41.95%. Annocript also enabled us to identify 3,133 putative long non-coding RNAs (lncRNA), whose definition was specified previously [64]. Due to the lack of the genomic sequence, these lncRNAs were not characterized further in this study. Among the annotated transcripts, a great majority encoded proteins, as a total of 32,406 transcripts corresponded to at least one protein in the UniRef90 database with the E-value lower than 5e10^-5^, and to 12,018 proteins in the SwissProt database. The transcriptome matched 4,891 protein domains in the CDD database, and 16 non-coding RNAs in Rfam. In view of taxonomy, the identified animal species that scored the most numerous closest hits to the *A*. *aranciacus* transcriptome in the UniRef90 database [75] to date was *S*. *purpuratus*, an echinoderm which exhibited 11,221 closest hits (34.2% of the cases), exceeding by far the second and third closest species: *Branchiostoma floridae* (2,046 hits) and *Nematostella vectensis* (529 hits) (Table 1). This reflects the fact that the animal species closer to *A*. *aranciacus*, e.g. starfish, have not been much presented in the annotated genetic sequence database of UniProt. It is also noteworthy that a great portion (37%) of the *A*. *aranciacus* transcriptome matched the so-called ‘unknown’ sequences in terms of the taxonomy of UniRef, for which unambiguous assignment of taxonomic origin was not possible mainly because the sequences contained more than one open reading frame.

**Table 1:** Comparison of the *A*. *aranciacus* reference transcriptome with the existing genetic sequence databases.

Species	Percentage
*Strongylocentrotus purpuratus*	34.2% (11,221)
*Branchiostoma floridae*	6.2% (2,046)
*Nematostella vectensis*	1.6% (529)
*Crassostrea gigas*	1.4% (470)
*Capitella teleta*	1.4% (456)
*Latimeria chalumnae*	1.0% (312)
*Danio rerio*	0.8% (258)
*Aplysia californica*	0.7% (231)
*Saccoglossus kowalevskii*	0.4% (145)
*Amphimedon queenslandica*	0.4% (145)
*Asterina pectinifera*	0.4% (144)
*Oreochromis niloticus*	0.4% (137)
*Xiphophorus maculatus*	0.3% (106)
*Xenopus tropicalis*	0.3% (106)
*Petromyzon marinus*	0.3% (100)

**Note:** A total of 32,783 annotated transcripts were analyzed with respect to the UniRef classification. Only the top 15 most frequent species exhibiting the closest matches to the entire transcriptome were presented in this table of NCBI taxonomy. Presented in parentheses are actual numbers of the different transcripts that scored the highest hits in the given reference species.

### Comparison of the stage-specific mRNA populations on the basis of expression profiles

**Fig 1:**
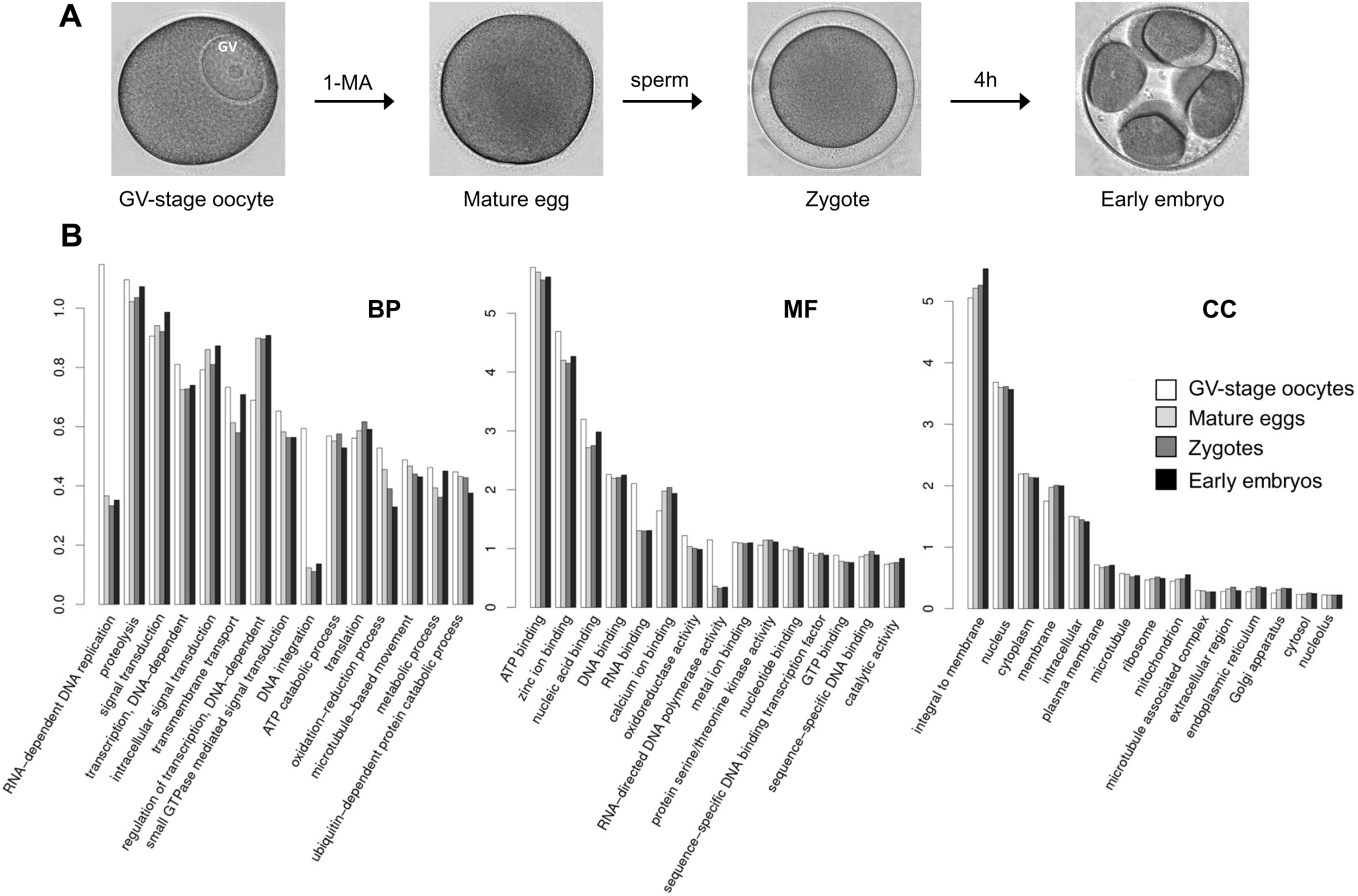
Comparison of the transcript population in the cells of the four different developmental stages based on the occurrence frequency of GO classes. (**A**) Morphology of the cells at the different developmental stages: GV-stage oocytes, mature eggs, zygotes (20 min post-fertilization), and the early embryos (4 h and 20 min post-fertilization). (**B**) Comparison of the four stages on the basis of the three GO domains: biological processes (BP), molecular functions (MF) and cellular components (CC). The numbers on the Y-axis represent the percentage of the transcripts belonging to the labeled category in reference to the net annotated transcript population at the given stage.

**Fig 2:**
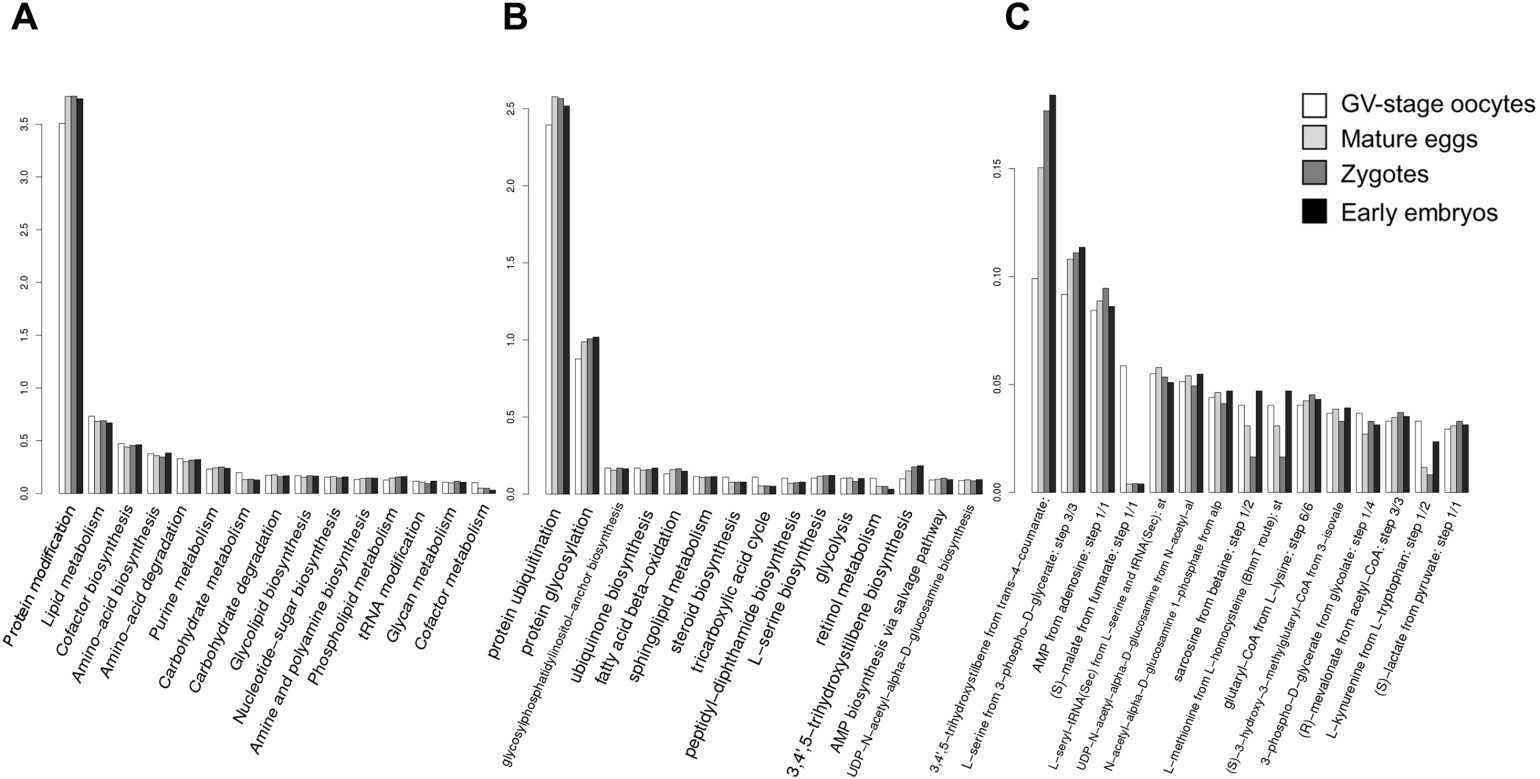
Comparison of the transcript population in the cells of the four different stages on the basis of pathway annotation. Transcripts were classified with respect to the metabolic pathways to which their deduced proteins contribute. The occurrence frequencies of the major pathways were plotted in reference to the net annotated transcripts in the cells of four stages (percentage, Y-axis). The three plots show the three different levels of pathways as in UniPathway: level 1 (**A**), level 2 (**B**), level 3 (**C**).

In an attempt to examine any shift in the mRNA population from eggs to early embryos, we have executed differential expression analyses of the transcripts at the different stages and annotated them on the basis of gene ontology (GO). When the count-based expression data from replicate samples (four animals) were analyzed by edgeR, it was estimated that the mRNA population hardly underwent abundance changes during the egg to zygote transition except for the 12 down-regulated transcripts (thus, 0.0186% of total transcripts). On the other hand, during the zygote to embryo transition (4h and 20 min post-fertilization), considerable changes were observed, as 2,137 out of 64,388 transcripts (3.3% of the total) were downregulated by more than 2-fold and 1,534 transcripts were upregulated (2.3%). When classified with respect to GO, the mRNA populations at the four stages covered a wide range of classes in terms of ‘biological processes’ (BP), ‘molecular functions’ (MF), and ‘cellular component’ (CC) (Fig 1). Apparently, the percentage of each functional GO class in the mRNA population underwent only marginal changes throughout meiotic maturation, fertilization, and cleavages. However, certain GO classes were conspicuously frequent in the cells of a specific developmental stage. For example, the transcripts classified as ‘DNA integration’ or ‘RNA-dependent DNA replication’ in the GO domain ‘biological processes’ (BP) were relatively more frequent in the GV-stage oocytes (Fig 1B, histogram BP). The proteins encoded by this class of transcripts may play a role in DNA replication or in retrotransposition process, respectively [76]. In line with this, when the expression profiles were compared in terms of MF, transcripts related to ‘RNA binding’ and ‘RNA-directed DNA polymerase activity’ were more frequently presented in the GV-stage oocytes in comparison with the rest (Fig 1B, histogram MF). In contrast, no marked difference was observed among the four different stages on the basis of the classification by CC (Fig 1B, histogram CC). As the great majority of the *A*. *aranciacus* transcripts encoded metabolic enzymes subserving specific physiological needs, we have compared the RNA population of the four stages on the basis of ‘pathway’ annotation [68]. As summarized in Fig 2, the transcriptome covered transcripts encoding enzymes of a wide variety of metabolic pathways, whose frequency of occurrence varied more than two orders of magnitude. For the enlisted 45 pathways, the mRNA populations of the four stages did not exhibit marked difference from one another. However, the GV-stage oocytes manifested more variety in the subset of transcripts encoding the enzymes involved in the ‘(S)-malate-from-fumarate’ pathway in comparison with the other stages (Fig 2C). This pathway refers to biochemical reactions involved in the Krebs cycle which regenerates NADH, the cell’s main reducing agent that donates electrons or produces ATP [77].

### Identification of the putative calcium signaling toolkit proteins

During the course of annotating the *A*. *aranciacus* transcriptome with BLAST, we were able to identify the transcripts encoding putative enzymes and ion channels that may play a role in the generation, propagation, or attenuation of intracellular Ca^2+^ waves. These are the enzymes that synthesize Ca^2+^-releasing second messengers such as InsP_3_ (phospholipase C), cyclic ADP-ribose and nicotinic acid adenine dinucleotide phosphate (ADP-ribosyl cyclase), or the ion channels and pumps that transport calcium ions across the membrane (Table 2). Out of the 38 transcripts tabulated in this category, 25 assembled sequences scored a hit coverage values greater than 85%, and many of them reached 95–100%. Even for the transcripts displaying moderate hit coverage values, the conserved regions were distributed throughout their entire length. For example, ryanodine receptor of *A*. *aranciacus* (5,161 AA) was most similar to that of sea urchin (*Hemicentrotus pulcherrimus*, 5,317 AA) [78] although the blast resulted in a coverage of 70%. Nonetheless, the global alignment between the two amino acid sequences using Clustal Omega indicated that the sequence similarity was maintained from the very start to the end with occasional short gaps. Hence, the great majority of the assembled sequences seemed to contain the entire coding region of the proteins of our interest.

**Table 2:** Transcripts encoding putative enzymes and ion channels involved in intracellular calcium signaling.

Transcripts	Annotation	Length (amino acid)	Coverage/ Identity	Top Hit Species	Accession No.	E-value
comp201526*	Inositol 1,4,5-trisphosphate receptor	2,736^a^	100%/86%	*Patiria pectinifera*	Q8WSR4	0.0
comp200930	Phospholipase C-beta4	1,243^b^	97%/71%	*Lytechinus pictus*	AAS55894	0.0
comp197619*	Phospholipase C-gamma1	2,160	100%/91%	*Patiria pectinifera*	AAR85355.1	0.0
comp197008	Phospholipase C-delta4	772^c^	100%/60%	*Strongylocentrotus purpuratus*	NP_001008790	0.0
comp191901	Phospholipase C-epsilon1	2,455	58%/45%	*Strongylocentrotus purpuratus*	XP_011680276	0.0
comp201603	Phospholipase C-eta2	2,150	38%/55%	Strongylocentrotus purpuratus	XP_011680765	0.0
comp202094*	Ryanodine Receptor	5,261^d^	70% / 68%	*Hemicentrotus pulcherrimus*	BAB84714	0.0
comp183145	ADP-ribosyl cyclase-like 1	343^e^	94%/43%	*Saccoglossus kowalevskii*	XP_002735899	2E-56
comp190002	ADP-ribosyl cyclase-like 2	320	98%/41%	Saccoglossus kowalevskii	XP_002735899	4E-20
comp188422	ADP-ribosyl cyclase-like 3	219	65%/46%	Saccoglossus kowalevskii	XP_002735899	4E-40
comp199516	Two-pore channel 1	915	89%/56%	*Strongylocentrotus purpuratus*	CBI63263	0.0
comp195246	Two-pore channel 2	830	99%/55%	*Strongylocentrotus purpuratus*	CBI63264	0.0
comp195231	Two pore calcium channel protein 1 isoform X1	1,022	99%/53%	*Strongylocentrotus purpuratus*	XP_011679870	0.0
comp198265	Sarco/endoplasmic reticulum calcium transporting ATPase (SERCA)	795	100%/81%	*Strongylocentrotus purpuratus*	XP_011663710	0.0
comp188433	Plasma membrane calcium transporting ATPase (PMCA)	1,141	99%/81%	*Strongylocentrotus purpuratus*	XP_011672381	0.0
comp181009	Sodium/calcium exchanger 3 (NCX) isoform X2	968	96%/70%	*Strongylocentrotus purpuratus*	XP_011661939	0.0
comp197721	Sodium/calcium exchanger 2	465	51%/63%	*Strongylocentrotus purpuratus*	XP_011683878	0.0
comp200471*	Sodium/calcium exchanger 3 isoform X6	801	97%/71%	*Strongylocentrotus purpuratus*	XP_794875	0.0
comp201718	Voltage-dependent calcium channel type A subunit alpha-1	1,255	51%/70%	*Strongylocentrotus purpuratus*	XP_011662956	0.0
comp196123	Voltage-dependent calcium channel subunit alpha-2/delta-1	1,084	99%/53%	*Strongylocentrotus purpuratus*	XP_011671023	0.0
comp199854	Voltage-dependent calcium channel subunit alpha-2/delta-2	741	58%/60%	*Lingula anatina*	XP_013386854	0.0
comp200305	Voltage-dependent calcium channel subunit alpha-2/delta-3	583	90%/49%	*Saccoglossus kowalevskii*	XP_006811294	1e-138
comp198886	Voltage-dependent calcium channel subunit alpha-2/delta-4	1,101	93%/41%	*Esox lucius*	XP_012994116	0.0
comp201327*	Voltage-dependent L-type calcium channel subunit beta-1	729	98%/66%	*Strongylocentrotus purpuratus*	XP_011661591	0.0
comp195452	Voltage-dependent T-type calcium channel subunit alpha-1G	2,395^f^	79%/49%	*Gallus gallus*	XP_015150974	0.0
comp162850	Sodium/potassium/calcium exchanger Nckx30C isoform X5 (NCKX)	660	92%/51%	*Agrilus planipennis*	XP_018318698	0.0
comp201917	Sodium/potassium/calcium exchanger 3	634	99%/57%	*Strongylocentrotus purpuratus*	XP_780438	0.0
comp192844	Sodium/potassium/calcium exchange CG1090 isoform X2	534	87%/44%	*Lingula anatina*	XP_013391924	6e-134
comp191999	Stromal interaction molecule 1 (STIM-1)	689	72%/62%	*Strongylocentrotus purpuratus*	XP_011666877	0.0
comp200692*	Orai-2-like	210	97%/73%	*Strongylocentrotus purpuratus*	XP_780791	9e-92
comp200190	Transient receptor potential cation channel (TRP) subfamily A1	1,393	100%/59%	*Strongylocentrotus purpuratus*	XP_797912	0.0
comp200408	TRP subfamily M3-like	1,090	76%/43%	*Saccoglossus kowalevskii*	XP_006816420	0.0
comp194828	TRP subfamily M2, isoform X6	1,475	87%/53%	*Strongylocentrotus purpuratus*	XP_011664677	0.0
comp200918	TRP subfamily M2	1,574	75%/51%	*Saccoglossus kowalevskii*	XP_006814003	0.0
comp191759	TRP subfamily V6	819	99%/48%	*Strongylocentrotus purpuratus*	XP_011668037	0.0
comp200620	TRP subfamily V5	759	89%/55%	*Strongylocentrotus purpuratus*	XP_786430	0.0
comp189572	Calcium channel flower homolog isoform X1	171	72%/52%	*Strongylocentrotus purpuratus*	XP_011671877	8e-40

* Acquisition of the composite sequence containing all the expected domains required higher coverage filtering.

^a-f^ Isoforms exist. The longest one was considered for BLAST analyses.

^a)^ An additional isoform exists lacking four amino acid residues (AA) 907–911.

^b)^ Three other isoforms exists lacking AA 499–532 to various extents.

^c)^ Two other isoforms exist; one lacks AA 151–157, the other lacks AA 151–157 and AA 541–542.

^d)^ An additional isoform exists that lacks AA 1389–1398 and contains a substitution (E1399K)

^e)^ Another isoform exists lacking AA 303–313 and contains a substitution (S301P)

^f)^ Three other isoforms exist; one lacks AA207-213 and contains a substitution (I206A), another lacks AA 2012–2025, and the third one has both of the two changes.

### Identification of cyclins and cyclin-dependent kinases

The cells of four developmental stages used in this study are of special interest for the study of cell cycle control. Starfish oocytes are arrested at the first prophase of meiotic cell cycle, whereas mature eggs have been just released from this block. Zygotes and early embryonic cells are to undergo rapid cycles of mitosis. As the key factors regulating the progression of cell cycle are cyclins and cyclin-dependent kinases, the cells of these stages are likely to be enriched with the mRNAs encoding these proteins. Indeed, annotation of the transcriptome led to identification of at least 13 distinct cyclins and as many cyclin-dependent kinases (Table 3). The tertiary structure of cyclin is characterized by the presence of two compact clusters of α-helices, so-called cyclin boxes, and the absence of ß sheets [79]. As expected, all putative cyclins identified in Table 3 contained the hallmark cyclin boxes and no ß sheet, as judged by Phyre2 analysis [80] (see https://figshare.com/s/de9aa8df2b0ef0e62dc1). It is noteworthy, however, that the putative cyclin K (comp200893) contained not only the two cyclin box folds near the N-terminus, but also an unusually long stretch of polypeptide chain that bears no sequence homology to the known protein domains in GenBank. Nonetheless, it was not unprecedented. Its homologous protein in *S*. *purpuratus* (XP_795740.3) was also intriguingly long for cyclin (816 AA) and had similar secondary structures. The unique polypeptide chains at the C-terminal side of these two putative cyclin K molecules were not similar to each other in their amino acid sequences. As to the cyclin-dependent kinase (CDK) family, all the putative enzymes enlisted in Table 3, except CDK-14, commonly contained all the characteristic domains such as cyclin interface, active site, ATP-binding sites, polypeptide substrate-binding sites, and an activation loop.

**Table 3:** Families of cyclin and cyclin-dependent kinase.

Transcripts	Annotation	Length (amino acid)	Coverage/Identity	Top Hit Species	Accession No.	E-value
comp196719	Cyclin A	446	99%/79%	*Patiria pectinifera*	BAA14010	0.0
comp190183	Cyclin B	403	100%/100%	*Astropecten aranciacus*	CAO99272	0.0
comp196609	Cyclin B3	447	100%/77%	*Marthasterias glacialis*	CBG91877	0.0
comp195049	Cyclin C	278^a^	98%/75%	*Saccoglossus kowalevskii*	XP_006817695	1e-151
comp195288	Cyclin D2	315	99%/61%	*Lingula anatina*	XP_013410672	1e-114
comp202146	Cyclin E	428	100%/81%	*Marthasterias glacialis*	CBG91878.1	0.0
comp196253	Cyclin F	594^b^	49%/49%	*Strongylocentrotus purpuratus*	XP_011666474	1e-120
comp190754	Cyclin G2	390	98%/46%	*Strongylocentrotus purpuratus*	XP_788820	1e-111
comp196371	Cyclin H	322	100%/57%	*Strongylocentrotus purpuratus*	XP_787341	6e-130
comp195488	Cyclin I	369	98%/51%	*Strongylocentrotus purpuratus*	XP_794154	1e-112
comp194056	Cyclin J	308	90%/52%	*Strongylocentrotus purpuratus*	XP_798281	1e-89
comp200893	Cyclin K	659	30%/81%	*Strongylocentrotus purpuratus*	XP_795740.3	4e-150
comp192356	Cyclin L1	582	78%/63%	*Strongylocentrotus purpuratus*	XP_790064	9e-175
comp196794	Cyclin-dependent kinase (CDK)1	306	100%/91%	*Patiria pectinifera*	BAA11477	0.0
comp185828	CDK2	300	100%/92%	*Patiria pectinifera*	BAH97197	0.0
comp194905	CDK5	296	100%/87%	*Saccoglossus kowalevskii*	XP_002732807	0.0
comp197484	CDK6	331	97%/65%	*Saccoglossus kowalevskii*	XP_002741831	2e-140
comp194115	CDK7	360^c^	89%/76%	*Anolis carolinensis*	XP_003216396	0.0
comp197368	CDK8	490	88%/80%	*Strongylocentrotus purpuratus*	XP_003728284	0.0
comp189294	CDK9	385	93%/75%	*Strongylocentrotus purpuratus*	XP_798269	0.0
comp196995	CDK10	400	96%/73%	*Strongylocentrotus purpuratus*	XP_797002.1	0.0
comp199997	CDK11B	696	47%/76%	*Saccoglossus kowalevskii*	XP_006823032	0.0
comp190838	CDK12	1,263	82%/52%	*Strongylocentrotus purpuratus*	XP_789337	0.0
comp201876	CDK14	478	96%/69%	*Strongylocentrotus purpuratus*	XP_011675680	0.0
comp191141	CDK17	371	100%/84%	*Strongylocentrotus purpuratus*	XP_011680140	0.0
comp190114	CDK20	340	100%/83%	*Saccoglossus kowalevskii*	XP_002741922	0.0

^a-c^ Isotypes exist. The longest one was considered for BLAST analyses.

^a)^ An additional isoform exists that lacks amino acid residues (AA) 270–278 and contains a substitution (S269R).

^b)^ An additional isoform exists lacking AA 446–476.

^c)^ An additional isoform exists varying at the C-terminus: AA 340–360 substituted by a shorter peptide GRLAKKLVF.

### Quantitation of the transcripts encoding the proteins of cyclin and CDK families

**Fig 3:**
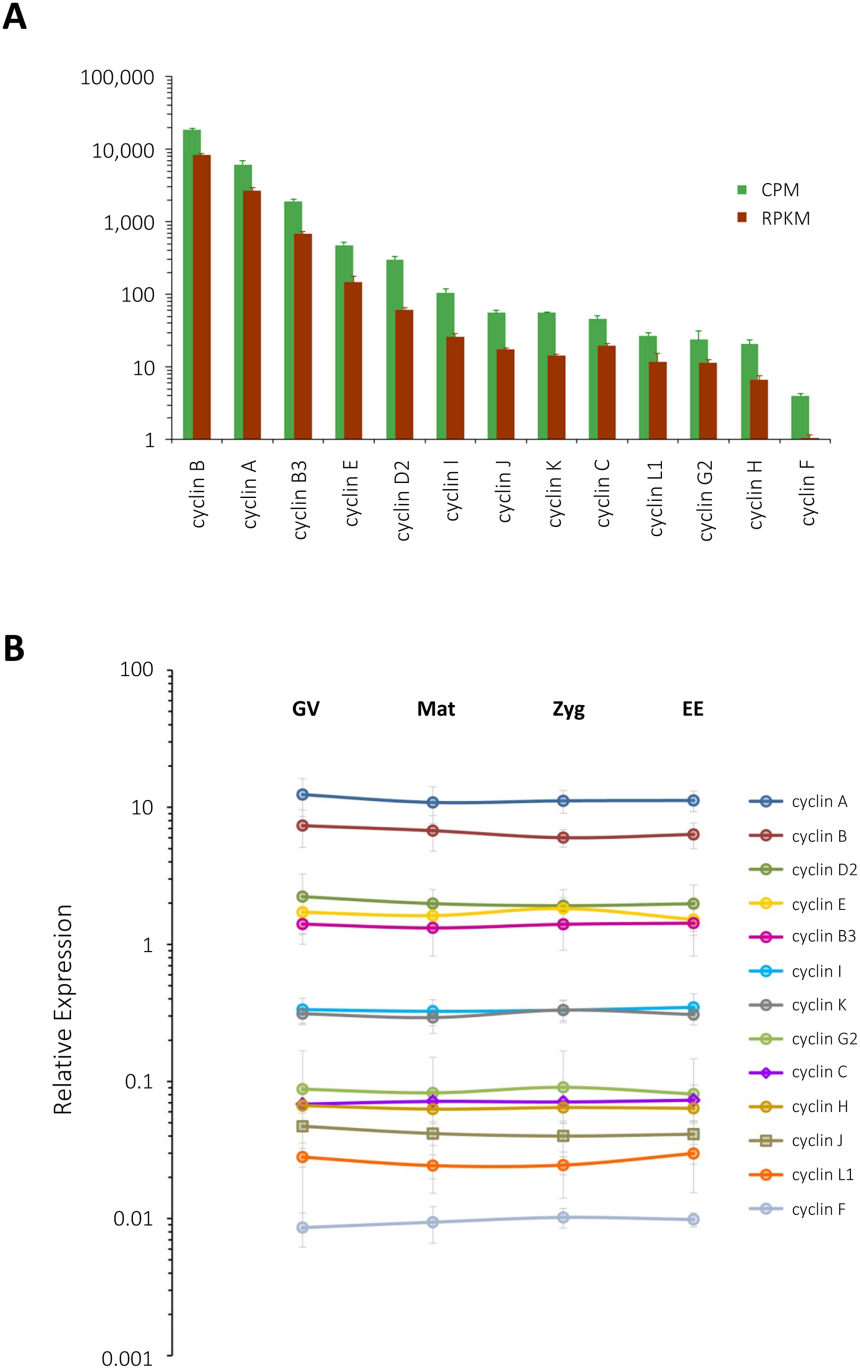
Quantitation of the transcripts encoding proteins of the cyclin family. (**A**) Abundance of the transcripts encoding distinct cyclins in mature eggs. Data were presented both in CPM (green bars) and in RPKM (brown bars). (**B**) Relative expression levels of cyclin family in the GV-stage oocytes (GV), mature eggs (Mat), zygotes (Zyg) and early embryos (EE) estimated by real-time qPCR. Data were normalized with the values of the internal control transcript and plotted on a logarithmic scale as described in Materials and methods. Histogram and data points with error bars indicate mean ± standard deviation (n = 4).

**Fig 4:**
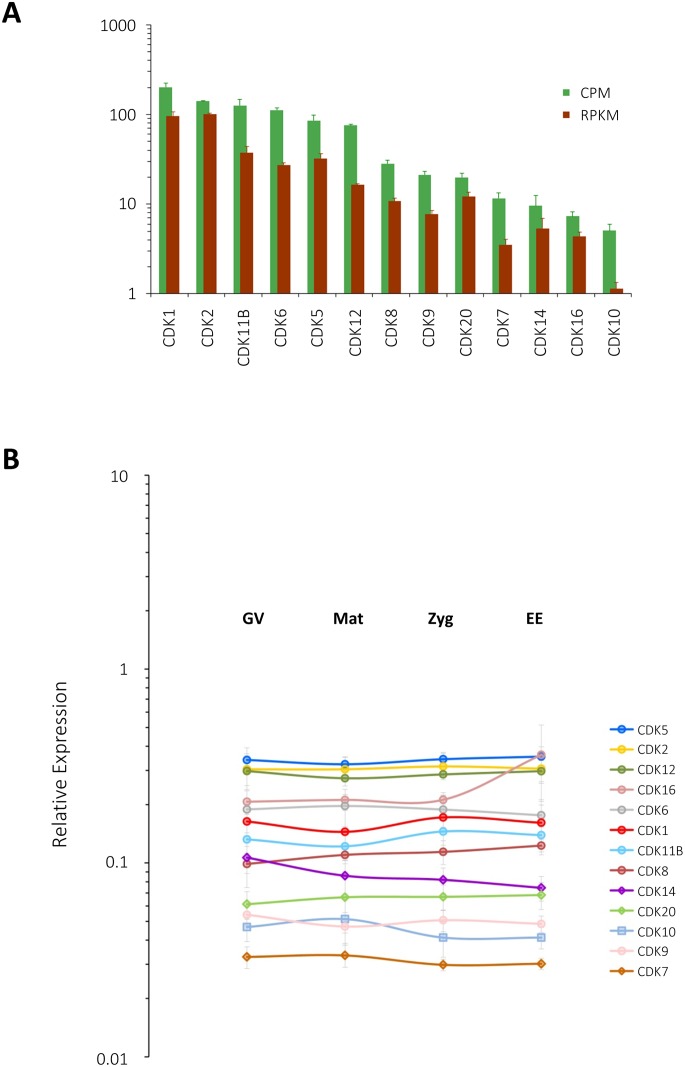
Quantitation of the transcripts encoding proteins of the CDK family. (**A**) Abundance of the transcripts encoding distinct members of the CDK family in mature eggs. Data were presented both in CPM (green bars) and in RPKM (brown bars). (**B**) Relative expression levels of CDK transcripts in GV-stage oocytes (GV), mature eggs (Mat), zygotes (Zyg) and early embryos (EE) estimated by real-time qPCR. Data were normalized with the values of the internal control transcript and plotted on a logarithmic scale as described in Materials and methods. Histogram and data points with error bars indicate mean ± standard deviation (n = 4).

As a means to assess quantitative validity of the transcriptome and to gain insights into the relevance of each member of cyclin and CDK families, RNA samples from the cells of four stages were subjected to real-time RT-PCR analyses by use of corresponding gene-specific primers (S1 Table), and the results were compared with the CPM (counts per million) values of each member of the cyclin and CDK families. Trinity assembly and edgeR analyses predicted that the transcripts of 13 distinct cyclin family members would greatly vary in their abundance. For example, it was estimated that cyclin B and A would be highly abundant in mature eggs, scoring 1.8x10^4^ CPM (thus, 1.8% of the total library) and 6.1x10^3^ CPM (0.6%), respectively. They were followed by cyclin B3, E, and D2, while cyclins L1, G2, H and F represented the rarest members of the cyclin family (Fig 3A, green bars). Thus, the CPM of cyclin members varied by four orders of magnitude. In large part, the CPM profile of cyclins is in agreement with the results of qPCR. When relative expression was presented as a ratio of each cyclin to the internal control transcript (splicing factor 5), cyclin A and B comprised the most abundant cyclins, followed by cyclins D2, E, and B3. Again, cyclin F was the least abundant, and cyclin H and L1 joined cyclin F as the rarest cyclin transcripts. Thus, the relative expression levels among cyclins varied at least by three orders of magnitude in qPCR (Fig 3B). However, there were also subtle disagreements between the two assessments. For example, cyclin B was three times as abundant as cyclin A in CPM, but the results of qPCR showed that cyclin A was more abundant than cyclin B by 60–85% throughout the stages, although the difference was statistically significant only in the zygotes (77%, P<0.05) and early embryos (85%, P<0.01). Likewise, when cyclins were ranked in the order of descending abundance in the two assessments, it appeared that cyclin D2 and G2 were slightly underestimated in CPM, while cyclin J was overestimated. These discrepancies might be attributable to the fact that the transcripts being compared varied in length, which was not formally reflected in CPM. Indeed, when transcript abundance was assessed in RPKM (Fig 3A, brown bars), cyclin C and G2 rose up by two steps and one, respectively, to assimilate to the qPCR data, but the rank order of more abundant transcripts (i.e. cyclins B to I) remained unchanged in comparison with CPM. The moderate discrepancy between the transcriptome and qPCR data cannot be ascribed to the potential differences in the primer efficiency among different genes, either, as the estimated primer efficiencies for all cyclins were virtually invariable (1.98 ± 0.08, n = 13) and identical to that of the internal control (1.95) [81]. It is also noteworthy that none of the cyclin transcripts underwent significant alterations of their relative abundance during meiotic progression, egg activation and cleavages (Fig 3B), suggesting that the noted periodic appearance of cyclins in starfish eggs and early embryos [82,83] during this period is likely to be regulated at the translational and post-translational levels. Unlike the cyclin family, transcripts of the CDK family were not much diverse in their abundance. For example, the difference between the most frequently encountered one (CDK1, 149 CPM) and the rarest (CDK10, 5 CPM) were merely 30-fold in mature eggs (Fig 4A, green bars). Reflecting nearly uniform distribution of their abundance, which was much lower than that of cyclin transcripts, the rank orders of CDK transcripts were often reversed in the qPCR analysis (Fig 4B). In particular, the results of qPCR indicated that CDK5 transcript was among the most abundant members of the CDK family together with CDK2 and CDK12 throughout these stages (Fig 4B). On the other hand, the transcript CDK1, encoding a component of MPF, was merely close to the median in qPCR and significantly lower than CDK5 in all these stages (P<0.05), although CDK1 was estimated to be the most abundant in the CPM analysis, and the second highest in RPKM (Fig 4A, brown bars). Nonetheless, in agreement with the transcriptome data (Fig 4A), transcripts encoding each member of the CDK family exhibited only a modest variability in qPCR analysis, as the most (CDK5) and the least (CDK7) abundant ones differed hardly beyond one order of magnitude (Fig 4B). The apparent increase (by 70%) of CDK16 during the zygote to early emryo transition in qPCR (Fig 4B) was not statistically significant (P = 0.3684), although the transcriptome data in RPKM predicted a 3-fold increase (P<0.001). Taken together, the results indicated that the assembled transcriptome was quantitative in general terms, but demonstrated compromised accuracy as judged by qPCR. The qPCR data corroborated the quantitative difference between two transcripts only when their abundance varied by 2–3 orders of magnitude. Thus, despite the general agreement between transcriptome and qPCR, it could be said that the transcriptome may not reliably predict more subtle differences in transcript abundance within one order of magnitude.

### Validation through selected genes

**Fig 5:**
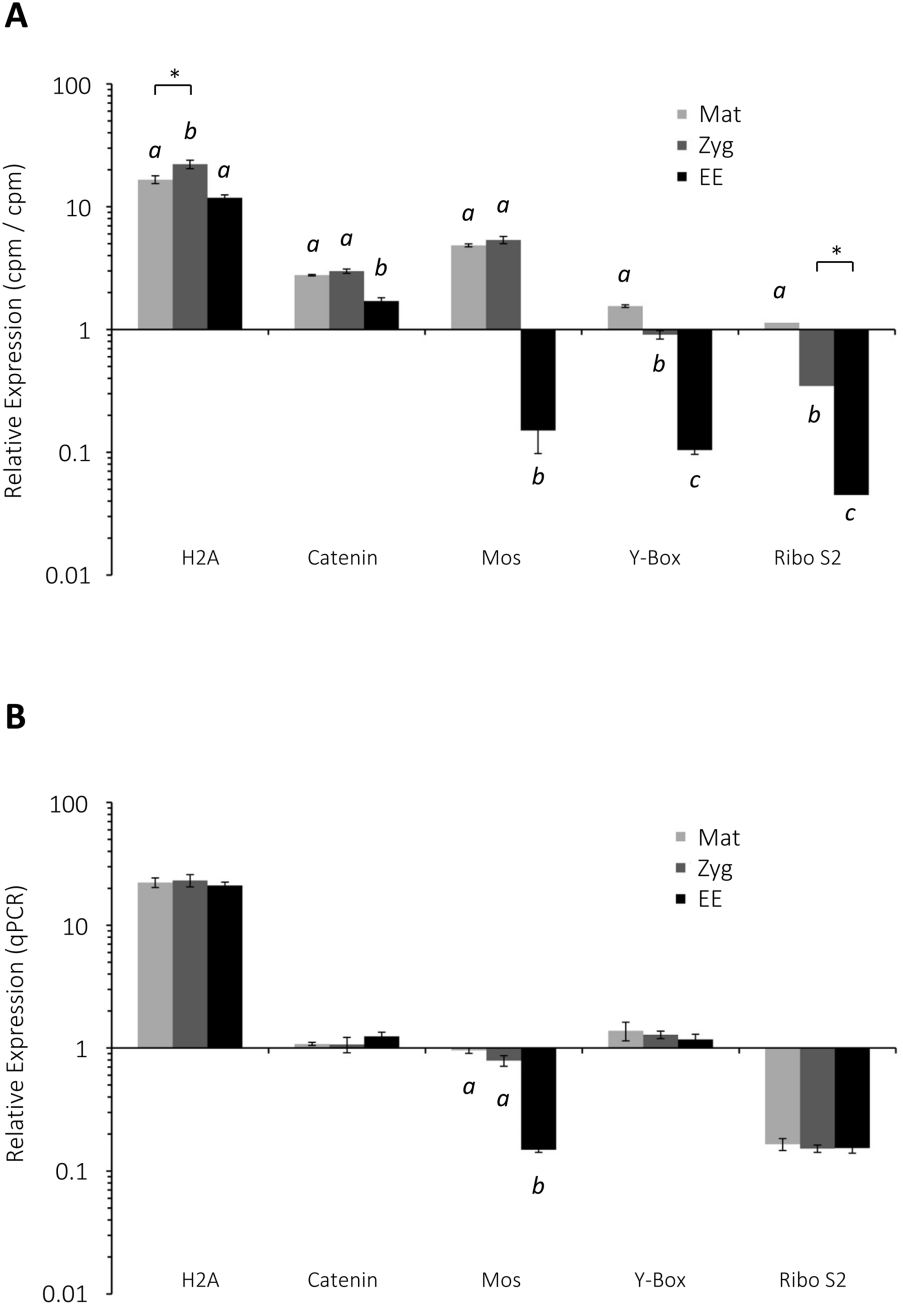
Comparison of the transcript levels of selected genes estimated by transcriptome statistics and real-time qPCR. (**A**) The cpm values of histone H2A (H2A), catenin ß, Mos, Y-Box transcription factor, and 40S ribosomal protein S2 (Ribo S2) were normalized with that of the internal control gene. (**B**) Quantitation of the same transcripts in mature eggs (Mat), zygotes (Zyg), and early embryos (EE) by real-time qPCR. The difference of the bars marked with distinct alphabetic characters (e.g. *a* versus *b*) is considered statistically significant. P<0.01 (*post hoc* test) in all cases except the ones indicated with the asterisks (P<0.05). Histograms with error bars indicate mean ± standard deviation.

For further quantitative assessment of the mRNA populations, we have selected five genes that manifested significant CPM changes during the course of egg activation and cleavages. As shown in Fig 5A, the transcript of histone H2A was over 10 times as numerous as the transcript of the control gene (splicing factor 5) in the count-based data of the transcriptome. Interestingly, the transcript count of H2A significantly increased (P<0.05) after fertilization (Fig 5A, H2A, asterisk), which was reminiscent of the transcriptional activation previously reported in the zygotes of sea urchin [84]. However, validation with qPCR agreed only to that H2A mRNA is over 10 times as abundant as the same control gene, but its modest increase during egg activation was not demonstrable by qPCR (Fig 5B, H2A). Similarly, the progressive decrease of the transcript encoding Y-Box transcription factor was also observed in qPCR, but the changes were not statistically significant. On the other hand, the quantitative data from Trinity assembly and qPCR did not match each other in the case of catenin ß and 40S ribosomal protein S2 (Ribo S2). Nonetheless, we were able to verify that the negative regulator of mitotic cell cycle, Mos, is significantly down-regulated as the zygote proceeds to cleavages (Fig 5B, Mos), which also makes sense physiologically. Hence, taken together with the qPCR data of cyclins and CDKs, we came to the conclusion that the assembled transcriptome was quantitative to a certain extent, but not precise enough to predict modest up- or down-regulation of individual genes with reliable accuracy. For this reason, we decided not to pursue further *in silico* quantitation of individual genes by use of the paradigms analyzing ‘enrichment’.

### Molecular phylogenetic analysis

**Fig 6:**
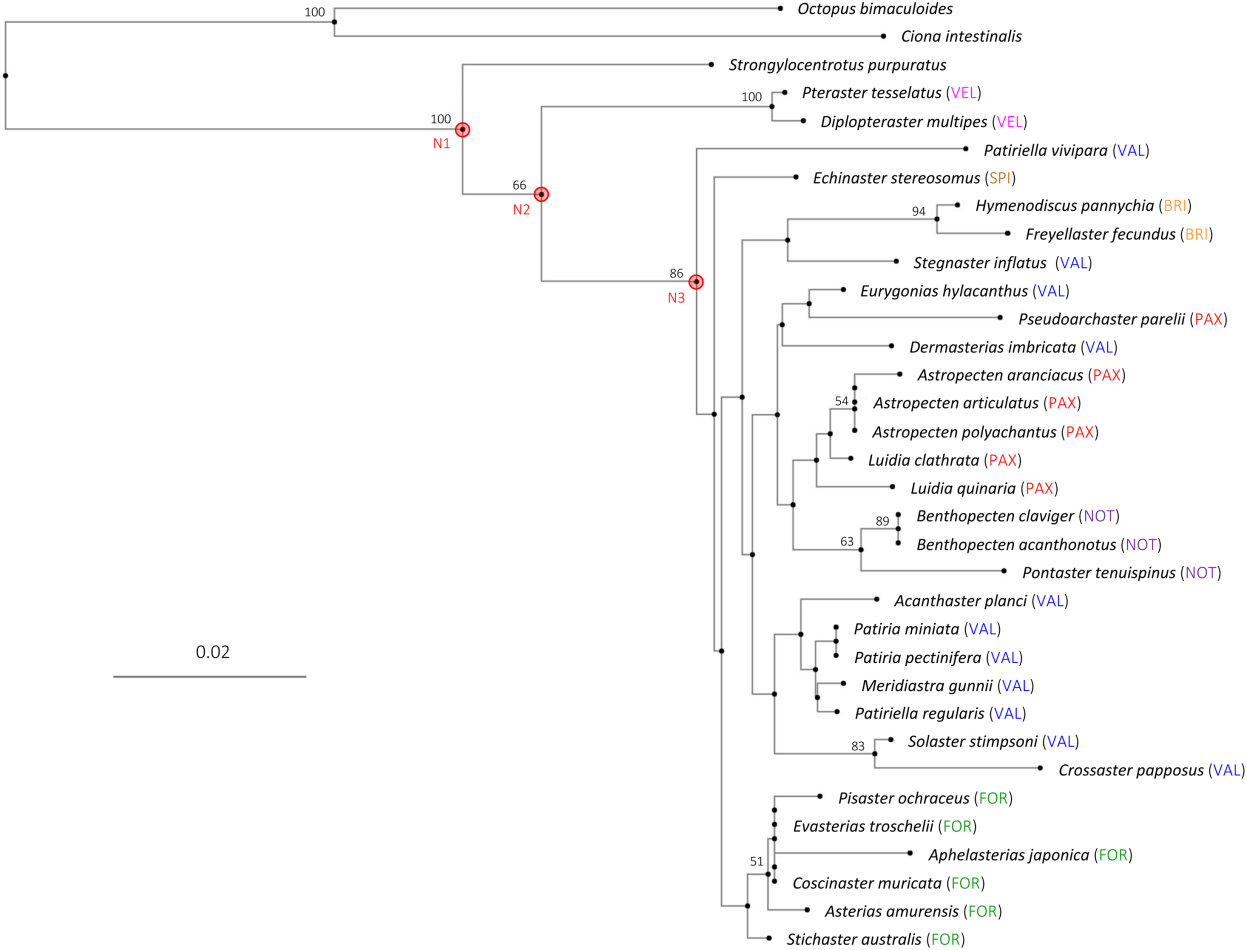
A phylogenetic tree of starfish based on the amino acid sequences of cytochrome oxidase subunit I (COS-I). The homologous amino acid sequences of uniform length were selected from the N-terminal coding region of the COI-I gene that was commonly available in 31 starfish species, and were subjected to multiple alignment and tree-building as described in Materials and Methods. Results of bootstrap analysis were indicated left to the corresponding nodes. For the sake of simplicity, bootstrap values below 50 were not indicated in the cladogram. The scale bar (0.1) for the branches shows the number of substitution per site. Abbreviations in parentheses represent taxonomic order of the species: BRI, Brisingida; FOR, Forcipulatida; NOT, Notomyotida; PAX, Paxillosida; SPI, Spinulosida; VAL, Valvatida; VEL, Velatida. N1, N2, and N3: key nodes discussed in the text.

We noted that the 8 most abundant transcripts in our transcriptome are all derived from the mitochondrial genome and encode enzymes regulating cell redox and bioenergetic reactions, e.g. cytochrome c oxidase subunit 1 (COI), 2 and 3; cytochrome b; NADH-ubiquinone oxidoreductase chain 1,4, and 5; ATP synthase subunit a. The DNA sequence of COI gene has been particularly useful for phylogenetic analyses [85–88]. To illustrate the utility of the transcriptome, we took this opportunity to compare COI, which was the most abundant mRNA in the transcriptome, with that of other starfish species, and built a phylogenetic tree on the basis of the strictly homologous region (Fig 6). When translated with the mitochondrial codons specific for echinoderms, a transcript from *A*. *aranciacus* (comp182578) encoded a protein whose deduced amino acid sequence (514 amino acid residues) matched the orthologues in most other starfish species with 99–90% identity. Unfortunately, the DNA sequence data for COI in starfish are scanty, and only partial sequences of the homologous gene (often not overlapping) were available for many species. Thus, we restricted our analysis to the region encoding the homologous peptides of uniform length near the N-terminus that are commonly available in most starfish species (211 amino acid residues ending with invariable sites, QHL). The collected amino acid sequences covered 7 orders of Asteroidea: Brisingida (2 species), Forcipulatida (6), Notomyotida (3), Paxillosida (6), Spinulosida (1), Valvatida (11) and Velatida (2). No species in the order Peripoda provided the homologous sequence in the corresponding region, and it was not included in our analysis. As referring points, instead, we included homologs from three outgroup species belonging to different phyla or class, i.e. *Ciona intestinalis* (phylum Chordata), *Octopus bimaculoides* (phylum Mollusca) and *S*. *purpuratus* (phylum Echinodermata, class Echinoidea), which shared with *A*. *aranciacus* about 67.5, 80.5 and 91.5% of sequence similarity at the amino acid level, respectively, within this homologous region. As expected, the gene region-specific phylogenetic tree constructed after multiple sequence alignment showed the two remote species *Octopus bimaculoides* and *Ciona intestinalis* first separated out with long branches (Fig 6). Then, at the next node, *sea urchin S*. *purpuratus* diverged from all the starfish species (N1, Fig 6). After that, two Velatida species *Diplopteraster multipes* and *Pteraster tesselatus* branched off from the rest of starfish (N2, Fig 6). At the following node N3, a Valvatida species *Patiriella vivipara* branched off from the rest. Up to this point, the phylogenetic relationships are supported by high or moderate confidence values of the bootstrap analysis (100 for N1, 66 for N2, and 83 for N3), but the following nodes exhibited relatively weak bootstrap values (<50) in the majority of cases, making it difficult to draw definitive conclusions on phylogenetic relationships. Nonetheless, they are not randomly mixed across the taxonomic boundaries, but often formed exclusive clades for the orders. For example, all the examined species of Forcipulatida comprised a monophyletic group (Fig 6). Although cautions must be taken in interpreting the contructed phylogenetic tree, the results of our analysis suggested that Velatida, and not Paxilosida, might be the most basal order of starfish.

## Discussion

*A*. *aranciacus* is a common starfish native to the Mediterranean Sea and the East Atlantic. Being encountered relatively easily, its systematic classification dates back to C. Linnaeus in 1758, and it has also been used as an excellent experimental animal model for biological researches ever since. To facilitate future studies of ours and others on reproduction, cell cycle, and intracellular Ca^2+^ signaling using starfish oocytes, eggs, and early embryos, we have assembled its first transcriptome, focusing on this relatively short developmental time frame. Our major goal in this study was to retrieve sequence data of the mRNAs encoding the key proteins playing their roles in the aforementioned biological processes of our interest. This approach was encouraged by the fact that oocytes and early embryos comprise mRNA populations of the richest variety [55]. The transcriptome assembled by RNA-seq methodology provided a collection of sequence data comprising 9.8x10^7^ nucleotides (= 68,218 contigs x 1,368 nucleotides on the average). Considering the species difference and some redundancy of the contigs, this number compares fairly well with the early estimation of the sequence complexity (the length of single copy nucleic acid sequence represented in the RNA population) of the sea urchin eggs estimated by the hybridization kinetics: 3.7x10^7^ nucleotides [55]. Annotating the transcriptome, we were able to identify 50.9% of these transcripts in the databases of SwissProt, UniRef, CCD, and Rfam. Most of them (98.9%) manifested E-values lower than 5e^-5^, an encouraging outcome on the identity of the annotated transcripts. The assembled transcriptome also exhibited acceptable results in the completeness analysis (CEGMA), as was reflected on the high coverage values ranging 85–100% in most transcripts encoding the putative enzymes and ion channels involved in intracellular Ca^2+^ signaling (Table 2) or the families of cyclin and CDKs (Table 3). The nucleic acid sequences of the transcripts encoding the key components of the specific cell signaling pathways are expected to enable us and others to develop molecular probes for the starfish cells and to perform comparative studies with other animal species. A comprehensive data set as such is only an example of the utility of our transcriptome.

In the list of the transcripts encoding Ca^2+^ signaling toolkit proteins (Table 2), we have identified 5 subtypes of the enzyme synthesizing InsP_3_ (PLC). The ζ isoform of PLC, which is present in mammalian sperm [43] as well as in the genomes of birds and bony fishes, was not found in the *A*. *aranciacus* transcriptome. To the best of our knowledge, PLC-ζ gene is also absent in the genomes of *S*. *purpuratus* and other echinoderm species mentioned in Introduction. Thus, its absence in the *A*. *aranciacus* transcriptome is not likely to be due to the negligible contribution of sperm’s RNA, if any, to the transcriptome of the zygote. Similar to sea urchin eggs [89], starfish eggs and early embryos contained transcripts encoding ion channels, pumps, and enzymes that cover nearly all known aspects of intracellular Ca^2+^ signaling: i) three ADP-ribosyl cyclases (cADPr and NAADP synthesis), ii) one InsP_3_ receptor; iii) one ryanodine receptor; iv) 3 two-pore channels (putative NAADP receptor); v) one SERCA, one PMCA, three Na^+^/Ca^2+^ exchangers, and three Na^+^/K^+^/Ca^2+^ exchangers (Ca^2+^ removal); vi) 7 voltage-gated Ca^2+^ channel subunits; vii) one Ca^2+^ channel flower homologue; viii) 6 TRP (transient receptor potential) channels. In addition, the transcriptome contained STIM-1 and orai-2, the putative components of a store-operated calcium channel. It would be of interest if the transcripts are also translated to functional proteins in the eggs and early oocytes, for it will exemplify operation of Ca^2+^ release-activated Ca^2+^ currents in excitable cells [90].

Cyclins are periodically synthesized and degraded at certain phases of cell cycle and thereby regulate enzyme activities of cyclin-dependent kinases that choreograph many aspects of mitosis and meiosis. For starfish, nucleic acid sequence of cyclin mRNA has been known only for cyclin A, B, B3, and E, but our study with *A*. *aranciacus* has revealed sequences of at least 9 other distinct cyclins being expressed in the eggs and early embryos of starfish (Table 3). Likewise, our study retrieved nucleic acid sequences of transcripts encoding 13 different putative CDKs. In theory, this might create enormously diverse cyclin/CDK pairings, but the functional combination of cyclin and CDK that regulate cell cycle is known to be restricted to a few specific pairings [91,92]. Thus, certain cyclin/CDK pairs carry out specialized roles in cell cycle control, while other members of cyclin and CDK families may not be directly involved in it [93]. Notably, all the CDKs of *A*. *aranciacus* being expressed by the early cleavage stage (Table 3) have their homologs in *S*. *purpuratus*, except for CDK6. Instead of it, *S*. *purpuratus* has CDK4, which was not present in the list of *A*. *aranciacus*. However, CDK4 is similar to CDK6 in that both molecules specifically pair with cyclin D, and that both CDKs may not be needed for cell cycle progression [93–95]. As cyclin D/CDK4 sustains normal development of sea urchin embryos beyond blastula stage [96], it would be interesting to see if CDK6 plays a similar role in the embryos of *A*. *aranciacus* or additional CDKs are expressed at a later developmental stage that was not addressed in our study.

Quantification of the relative gene expression levels by real-time qPCR confirmed that cyclin A and B are the two most abundant members of cyclin family in the oocytes, eggs, zygotes, and early embryos of *A*. *aranciacus* (Fig 3B). Both cyclin A and B can bind to CDK1 to regulate its enzyme activity [97], and studies in starfish have shown that cyclin B/CDK1 is almost exclusively accountable for the MPF activity during the meiotic progression of the oocytes (maturation). Instead, cyclin A/CDK1 activity is negligible during meiotic maturation, but becomes predominant at mitosis during early cleavages [83]. It is because cyclin A protein is nearly undetectable until the first cleavage, while cyclin B is newly synthesized in the maturing oocytes and degraded at first meiotic anaphase [83,98]. Hence, our finding that mRNA levels of the cyclin and CDK families remain unchanged during the period of meiotic cycle and the first few cleavages (Fig 3B) indicate that oscillation of cyclins is not regulated at the transcriptional level.

In this context, it should be remembered that the mRNAs stockpiled in the eggs and early embryos are mostly metabolic provisions for future use during development, and do not necessarily reflect the current composition of the protein population of the cells. Translation activity remains at the basal level in starfish oocytes, but the overall activity of protein synthesis is evidently enhanced by the time of GVBD when expression of some selected proteins such as cyclin B is overtly enhanced [24,56,98,99]. After fertilization, the rate of *de novo* protein synthesis is accelerated with a considerable time lag for most animal species [24,100–105], as mRNAs get unmasked, polyadenylated, or engaged with active polysomes [106–108]. Other post-transcriptional controls such as polyadenylation or partial degradation of the transcripts during meiotic maturation and fertilization may also contribute to translatability of the mRNAs [109,110]. Hence, quantitative characterization of the transcriptome alone does not provide sufficient information on biochemical activities of the eggs and early embryos. In this regard, qualitative and spatial controls of mRNA should also be carefully examined, and be complemented by other approaches such as proteomics in future studies [89,111].

During the periods of meiotic maturation and fertilization, the maternal mRNA population might change as a result of RNA synthesis and degradation. In sea urchin, *de novo* transcription seems to take place in the pronucleus of mature eggs, and presumably also in the male pronuclei of the zygotes [112,113]. In the clam oocytes and early embryos, on the other hand, the competent mRNA population was virtually unchanged during this transition period, as judged by the results of the cell-free translation system [103]. In our study, despite the great diversity of the mRNA population in the oocytes, eggs and early embryos, the profiles of the mRNA population apparently did not undergo a marked shift except for the frequency changes of the transcripts belonging to few GO classes (Fig 1) or a metabolic pathway (Fig 2). Nonetheless, the analyses of the assembled transcriptome predicted that a number of transcripts may undergo considerable changes in their abundance during the period of maternal to zygote transition. However, validation of the transcriptome data with real-time qPCR indicated that the estimations by the two methods agreed with each other only on a large scale, and that the differences smaller than 10-fold were often difficult to resolve (Fig 5). This discrepancy may stem from the facts that: *i*) digital gene expression levels by RNA-seq displays wider dynamic range than qPCR does [114], *ii*) the RNA samples for RNA-seq and qPCR were taken from different breeding seasons for a more rigorous verification (biological replicates rather than technical replicates), *iii*) sequencing depth may have not been sufficient to minimize the variability of *in silico* analysis [115], or iv) efficiency of reverse transcription might have been substantially variable among transcripts of different genes. The possibilities of variable contributions from genomic DNA contamination or from the batch effect of assembled sequences were all ruled out by the control experiments using RNAse A (S1 Fig) and cluster analysis (S2 Fig), respectively. At any rate, we have confirmed that the level of the transcript encoding a serine/threonine-protein kinase, Mos, is significantly reduced after a few rounds of cleavage (Fig 5). In view of the fact that Mos is a negative regulator of cell cycle [116,117], its significant reduction in transcript number during the rapid rounds of mitosis makes a perfect sense.

The phylogeny of starfish has been highly controversial, especially for the placement of Paxillosida to which *A*. *aranciacus* belongs [59]. The ordinal classification of starfish has mainly relied on anatomical and embryological characters (e.g. morphology of pedicellarie and spines, eversion of cardiac stomach, and developmental passage through brachiolaria stage, etc.) because DNA sequence information is largely lacking for most species [57,118]. The genome of mitochondrial DNA has been recognized as a useful source of phylogenetic analysis for the cases in which phylogenetic distance is relatively small [119]. Here, we considered 6 Paxillosidan species falling into three families (i.e. Luidiidae, Astropectinidae, and Pseudarchasteridae) along with 25 other starfish species. Although the length of homologous region is relatively short and the taxa sampling is sparse due to the limited availability of DNA sequences, our analysis with the deduced amino acid sequences revealed approximate inter-relationship of orders. Whereas previous molecular trees often suggested Paxillosida as the most basal order in the phylogeny of starfish [120,121], our results rather suggested that two Velatidan species were basal to all the rest starfish species (Fig 6). This discrepancy may in part arise from the fact that the aforementioned studies did not include the two Velatidan species in their analyses. Our data placing Paxillosida as a derived order are in line with more recent studies utilizing 13 protein-coding genes in the mitochondrial genome [122].

In summary, we have described the utility of the first transcriptome that was assembled from *A*. *aranciacus*. Despite the inevitable limitations stemming from the lack of genomic sequences and the relatively short developmental time frame, the annotated transcriptome is likely to provide useful information in designing molecular approaches to a number of biological research topics that utilize starfish.

## Supporting information

S1 Table Gene-specific primer sequences utilized in RT-PCR experiments. (XLSX)

S1 Fig Amplification of cyclin A from RNA samples by RT-PCR. Biological replicates of RNA samples representing each stage, i.e. GV oocytes (GV), mature eggs (M), Zygotes (Z) and early embryos (EE), were pooled together and converted to cDNA. As a negative control, aliquots of RNA were pretreated with DNAse-free RNAse prior to cDNA synthesis, as described previously [70]. While cyclin A primers (S1 Table) produced abundant amplicons in RT-PCR after saturating number of cycles (= 40), no detectable amplicons were present in the PCR product from the samples pretreated with RNAse, suggesting that virtually all PCR products originated from RNA, not DNA. Lanes. L, 100 bp DNA ladder; (+) RNAse-pretreated samples; (-) Samples without RNA pretreatment.
(TIF)

S2 Fig A plot of Multidimensional Scaling analysis for the read counts from the biological replicates of RNA samples. The FPKM (Fragments Per Kilobase of transcript per Million mapped reads) read counts were clustered using the cmdscale function in R. It is evident that the biological replicates of each stage were converged in a restricted area, but were appreciably separated from the converged replicates of other stages. Reflecting the little changes of gene expression between Mature eggs (Mat) and Zygotes (Zyg, fertilized eggs), the converged area defined by M1-M4 was relatively close to that of F1-F4. The data from the GV-stage were not included in this cluster analysis because they were processed as pooled samples.
(TIF)

## References

[1] Smith AB, Jell PA. Cambrian edrioasteroids from Australia and the origin of starfishes. Memoirs of the Queensland Museum 1990;28: 715–778.

[2] Hinman VF, Nguyen AT, Cameron RA, Davidson EH. Developmental gene regulatory network architecture across 500 million years of echinoderm evolution. Proc Natl Acad Sci U S A. 2003;100: 13356–13361. doi: 10.1073/pnas.2235868100 14595011 10.1073/pnas.2235868100 PMC263818

[3] Mah CL, Blake DB. Global diversity and phylogeny of the Asteroidea (Echinodermata). PLoS One. 2012;7: e35644 doi: 10.1371/journal.pone.0035644 22563389 10.1371/journal.pone.0035644 PMC3338738

[4] Paine RT. A short-term experimental investigation of resource partitioning in a New Zealand rocky intertidal habitat. Ecology. 1971;52: 1096–1106.

[5] Ross DJ, Johnson CR, Hewitt CL, Ruiz GM. Interaction and impacts of two introduced species on a soft-sediment marine assemblage. Mar. Biol. 2004;144: 747–756.

[6] Kayal M, Vercelloni J, Lison de Loma T, Bosserelle P, Chancerelle Y, Geoffroy S, et al Predator crown-of-thorns starfish (Acanthaster planci) outbreak, mass mortality of corals, and cascading effects on reef fish and benthic communities. PLoS One. 2012;7: e47363 doi: 10.1371/journal.pone.0047363 23056635 10.1371/journal.pone.0047363 PMC3466260

[7] Lawrence JM. Arm loss and regeneration in Asteroidea (Echinodermata) In: Scalera-Liaci L, Canicattı C, editors. Echinoderm research. Rotterdam: Balkema; 1992 pp 39–52.

[8] Chia FS, Oguro C, Komatsu M. Sea-star (asteroid) development. Oceanogr. Mar. Biol Annu. Rev. 1993;31: 223–257.

[9] Tyler A, Monroy A, Kao CY, Grundfest H. Membrane potential and resistance of the starfish egg before and after fertilization. Biol Bull. 1956;111: 153–177.

[10] Miyazaki SI, Ohmori H, Sasaki S. Action potential and non-linear current-voltage relation in starfish oocytes. J Physiol. 1975;246: 37–54. 1169319 10.1113/jphysiol.1975.sp010879 PMC1309403

[11] Dale B, De Santis A, Hoshi M. Membrane response to 1-methyladenine requires the presence of the nucleus. Nature. 1979;282: 89–90. 503193 10.1038/282089a0

[12] Chiba K, Kado RT, Jaffe LA. Development of calcium release mechanisms during starfish oocyte maturation. Dev Biol. 1990;140: 300–306. 2373255 10.1016/0012-1606(90)90080-3

[13] Santella L, Kyozuka K. Reinitiation of meiosis in starfish oocytes requires an increase in nuclear Ca^2+^. Biochem Biophys Res Commun. 1994;203: 674–680. doi: 10.1006/bbrc.1994.2235 8074721 10.1006/bbrc.1994.2235

[14] Stricker SA, Centonze VE, Melendez RF. Calcium dynamics during starfish oocyte maturation and fertilization. Dev Biol. 1994;166: 34–58. doi: 10.1006/dbio.1994.1295 7958457 10.1006/dbio.1994.1295

[15] Stricker SA. Time-lapse confocal imaging of calcium dynamics in starfish embryos. Dev Biol. 1995;170: 496–518. doi: 10.1006/dbio.1995.1232 7649379 10.1006/dbio.1995.1232

[16] Santella L, Nusco GA, Lim D. Calcium and Calcium-Linked Second Messengers are main Actors in the Maturation and Fertilization of Starfish Oocytes In: Lee HC, editor. Cyclic ADP-Ribose and NAADP: Structures, Metabolism and Functions. Boston: Keuwer Academic Publishers; 2002 pp. 381–396.

[17] Santella L, Puppo A, Chun JT. The role of the actin cytoskeleton in calcium signaling in starfish oocytes. Int J Dev Biol. 2008;52: 571–584. doi: 10.1387/ijdb.072560ls 18649271 10.1387/ijdb.072560ls

[18] Lim D, Ercolano E, Kyozuka K, Nusco GA, Moccia F, Lange K, et al The M-phase-promoting factor modulates the sensitivity of the Ca^2+^ stores to inositol 1,4,5-trisphosphate via the actin cytoskeleton. J Biol Chem. 2003;278: 42505–42514. doi: 10.1074/jbc.M301851200 12867432 10.1074/jbc.M301851200

[19] Lénárt P, Bacher CP, Daigle N, Hand AR, Eils R, Terasaki M, et al A contractile nuclear actin network drives chromosome congression in oocytes. Nature. 2005;436: 812–818. doi: 10.1038/nature03810 16015286 10.1038/nature03810

[20] Kyozuka K, Chun JT, Puppo A, Gragnaniello G, Garante E, Santella L. Actin cytoskeleton modulates calcium signalling during the maturation of starfish oocytes. Dev. Biol. 2008;320: 426–435. doi: 10.1016/j.ydbio.2008.05.549 18599031 10.1016/j.ydbio.2008.05.549

[21] Chun JT, Puppo A, Vasilev F, Gragnaniello G, Garante E, Santella L. The biphasic increase of PIP2 in the fertilized eggs of starfish: new roles in actin polymerization and Ca^2+^ signaling. PLoS One. 2010;5: e14100 doi: 10.1371/journal.pone.0014100 21124897 10.1371/journal.pone.0014100 PMC2990714

[22] Limatola N, Chun JT, Kyozuka K, Santella L. Novel Ca^2+^ increases in the maturing oocytes of starfish during the germinal vesicle breakdown. Cell Calcium. 2015;58: 500–510. doi: 10.1016/j.ceca.2015.08.002 26318405 10.1016/j.ceca.2015.08.002

[23] Kanatani H, Shirai H, Nakanishi K, Kurokawa T. Isolation and identification on meiosis inducing substance in starfish Asterias amurensis. Nature. 1969;221: 273–274. 5812580 10.1038/221273a0

[24] Houk MS, Epel D. Protein synthesis during hormonally induced meiotic maturation and fertilization in starfish oocytes. Dev Biol. 1974;40: 298–310. 4430410 10.1016/0012-1606(74)90132-8

[25] Moreau M, Guerrier P, Doree M, Ashley CC. Hormone-induced release of intracellular Ca^2+^ triggers meiosis in starfish oocytes. Nature 1978;272: 251–253. 556362 10.1038/272251a0

[26] Schroeder TE. Microfilament-mediated surface change in starfish oocytes in response to 1-methyladenine: implications for identifying the pathway and receptor sites for maturation-inducing hormones. J Cell Biol. 1981;90: 362–371. 6270153 10.1083/jcb.90.2.362 PMC2111864

[27] Meijer L, Pondaven P, Tung HY, Cohen P, Wallace RW. Protein phosphorylation and oocyte maturation. II. Inhibition of starfish oocyte maturation by intracellular microinjection of protein phosphatases 1 and 2A and alkaline phosphatase. Exp Cell Res. 1986;163: 489–499. 3007183 10.1016/0014-4827(86)90079-0

[28] Santella L, De Riso L, Gragnaniello G, Kyozuka K. Cortical granule translocation during maturation of starfish oocytes requires cytoskeletal rearrangement triggered by InsP_3_-mediated Ca^2+^ release. Exp Cell Res. 1999;248:567–574. doi: 10.1006/excr.1999.4425 10222148 10.1006/excr.1999.4425

[29] Wessel GM, Brooks JM, Green E, Haley S, Voronina E, Wong J, et al The biology of cortical granules. Int Rev Cytol. 2001;209: 117–206. 11580200 10.1016/s0074-7696(01)09012-x

[30] Chun JT, Santella L. The actin cytoskeleton in meiotic maturation and fertilization of starfish eggs. Biochem Biophys Res Commun. 2009;384: 141–143. doi: 10.1016/j.bbrc.2009.04.087 19393631 10.1016/j.bbrc.2009.04.087

[31] Lim D, Kyozuka K, Gragnaniello G, Carafoli E, Santella L. NAADP^+^ initiates the Ca^2+^ response during fertilization of starfish oocytes. FASEB J. 2001;15: 2257–2267. doi: 10.1096/fj.01-0157com 11641253 10.1096/fj.01-0157com

[32] Labbé JC, Capony JP, Caput D, Cavadore JC, Derancourt J, Kaghad M, et al MPF from starfish oocytes at first meiotic metaphase is a heterodimer containing one molecule of cdc2 and one molecule of cyclin B. EMBO J. 1989;8: 3053–3058. 2531073 10.1002/j.1460-2075.1989.tb08456.x PMC401383

[33] Meijer L, Guerrier P. Maturation and fertilization in starfish oocytes. Int Rev Cytol. 1984;86:129–196. 6423562 10.1016/s0074-7696(08)60179-5

[34] Santella L, Limatola N, and Chun JT. Actin cytoskeleton and fertilization in starfish eggs In: Sawada H, Inoue N, Iwano M, editors. Sexual Reproduction in Animals and Plants. Tokyo: Springer Verlag; 2014 pp. 141–155.

[35] Trimmer JS, Vacquier VD. Activation of sea urchin gametes. Annu Rev Cell Biol. 1986;2: 1–26. doi: 10.1146/annurev.cb.02.110186.000245 3548763 10.1146/annurev.cb.02.110186.000245

[36] Krauchunas AR, Wolfner MF (2013) Molecular changes during egg activation. Curr Top Dev Biol. 102:267–292. doi: 10.1016/B978-0-12-416024-8.00010-6 23287037 10.1016/B978-0-12-416024-8.00010-6 PMC3931425

[37] Santella L, Limatola N, Chun JT. Calcium and actin in the saga of awakening oocytes. Biochem Biophys Res Commun. 2015;460: 104–113. doi: 10.1016/j.bbrc.2015.03.028 25998739 10.1016/j.bbrc.2015.03.028

[38] Jaffe LA, Giusti AF, Carroll DJ, Foltz KR. Ca^2+^ signalling during fertilization of echinoderm eggs. Semin Cell Dev Biol. 2001;12: 45–51. doi: 10.1006/scdb.2000.0216 11162746 10.1006/scdb.2000.0216

[39] Jaffe L. On the conservation of fast calcium wave speeds. Cell Calcium. 2002;32: 217–229. 12379182 10.1016/s0143416002001574

[40] Santella L, Vasilev F, Chun JT. Fertilization in echinoderms. Biochem Biophys Res Commun. 2012;425: 588–594. doi: 10.1016/j.bbrc.2012.07.159 22925679 10.1016/j.bbrc.2012.07.159

[41] Chun JT and Santella L. Intracellular Calcium Waves In: Lennarz WJ, Lane MD, editors. The Encyclopedia of Biological Chemistry, Vol. 2 Academic Press: Waltham; 2013 pp. 640–647.

[42] Swann K, Lai FA. Egg Activation at Fertilization by a Soluble Sperm Protein. Physiol Rev. 2016;96: 127–149. doi: 10.1152/physrev.00012.2015 26631595 10.1152/physrev.00012.2015

[43] Rhee SG. Regulation of phosphoinositide-specific phospholipase C. Annu Rev Biochem. 2001;70: 281–312. doi: 10.1146/annurev.biochem.70.1.281 11395409 10.1146/annurev.biochem.70.1.281 PMC4781088

[44] Saunders CM, Larman MG, Parrington J, Cox LJ, Royse J, Blayney LM, et al PLC zeta: a sperm-specific trigger of Ca^2+^ oscillations in eggs and embryo development. Development. 2002;129: 3533–3544. 12117804 10.1242/dev.129.15.3533

[45] Sodergren E, et al in Sea Urchin Genome Sequencing Consortium. The genome of the sea urchin *Strongylocentrotus purpuratus*. Science. 2006;314: 941–952. doi: 10.1126/science.1133609 17095691 10.1126/science.1133609 PMC3159423

[46] Lee HC. Mechanisms of calcium signaling by cyclic ADP-ribose and NAADP. Physiol Rev. 1997;77: 1133–1164. 9354813 10.1152/physrev.1997.77.4.1133

[47] Galione A, White A, Willmott N, Turner M, Potter BV, Watson SP. cGMP mobilizes intracellular Ca^2+^ in sea urchin eggs by stimulating cyclic ADP-ribose synthesis. Nature. 1993;365: 456–459. doi: 10.1038/365456a0 7692303 10.1038/365456a0

[48] Santella L, Lim D, Moccia F. Calcium and fertilization: the beginning of life. Trends Biochem Sci. 2004;29:400–408. doi: 10.1016/j.tibs.2004.06.009 15362223 10.1016/j.tibs.2004.06.009

[49] Nusco GA, Lim D, Sabala P, Santella L. Ca^2+^ response to cADPr during maturation and fertilization of starfish oocytes. Biochem Biophys Res Commun 2002;290: 1015–1021. doi: 10.1006/bbrc.2001.6286 11798176 10.1006/bbrc.2001.6286

[50] Santella L and Chun JT. Calcium Signaling by Cyclic ADP-Ribose and NAADP In: Lennarz WJ, Lane MD, editors. The Encyclopedia of Biological Chemistry, Vol. 1 Waltham: Academic Press; 2013 pp. 331–336.

[51] Lim D, Lange K, Santella L. Activation of oocytes by latrunculin A. FASEB J. 2002;16: 1050–1056. doi: 10.1096/fj.02-0021com 12087066 10.1096/fj.02-0021com

[52] Puppo A, Chun JT, Gragnaniello G, Garante E, Santella L. Alteration of the cortical actin cytoskeleton deregulates Ca^2+^ signaling, monospermic fertilization, and sperm entry. PLoS One. 2008;3: e3588 doi: 10.1371/journal.pone.0003588 18974786 10.1371/journal.pone.0003588 PMC2570615

[53] Berridge MJ, Lipp P, Bootman MD. The versatility and universality of calcium signalling. Nat Rev Mol Cell Biol. 2000;1:11–21. doi: 10.1038/35036035 11413485 10.1038/35036035

[54] Chun JT, Limatola N, Vasilev F, Santella L. Early events of fertilization in sea urchin eggs are sensitive to actin-binding organic molecules. Biochem Biophys Res Commun. 2014;450: 1166–1174. doi: 10.1016/j.bbrc.2014.06.057 24960199 10.1016/j.bbrc.2014.06.057

[55] Galau GA, Klein WH, Davis MM, Wold BJ, Britten RJ, et al Structural gene sets active in embryos and adult tissues of the sea urchin. Cell. 1976;7: 487–505. 986248 10.1016/0092-8674(76)90200-2

[56] Brandhorst BP. Informational content of the echinoderm egg. Dev Biol. 1985;1: 525–576. 10.1007/978-1-4615-6814-8_12 2481472

[57] Blake DB. Classification and phylogeny of post-Paleozoic sea stars (Asteroidea: Echinodermata). J Nat Hist. 1987;21: 481–528.

[58] Janies DA, Voight JR, Daly M. Echinoderm phylogeny including Xyloplax, a progenetic asteroid. Syst Biol. 2011;60: 420–438. doi: 10.1093/sysbio/syr044 21525529 10.1093/sysbio/syr044

[59] Gale AS. Phylogeny of the Astroidea In: Lawrence JM, editor. Starfish: biology andecology of the Asteroidea. Baltimore: The Jons Hopkins University Press; 2013 pp. 3–14.

[60] Bolger AM, Lohse M, Usadel B. Trimmomatic: a flexible trimmer for Illumina sequence data. Bioinformatics. 2014;30: 2114–2120. doi: 10.1093/bioinformatics/btu170 24695404 10.1093/bioinformatics/btu170 PMC4103590

[61] Grabherr MG, Haas BJ, Yassour M, Levin JZ, Thompson DA, Amit I, et al Full-length transcriptome assembly from RNA-Seq data without a reference genome. Nat Biotechnol. 2011;29: 644–652. doi: 10.1038/nbt.1883 21572440 10.1038/nbt.1883 PMC3571712

[62] Li W, Godzik A. Cd-hit: a fast program for clustering and comparing large sets of protein or nucleotide sequences. Bioinformatics. 2006;22: 1658–1689. doi: 10.1093/bioinformatics/btl158 16731699 10.1093/bioinformatics/btl158

[63] Parra G, Bradnam K, Korf I. CEGMA: a pipeline to accurately annotate core genes in eukaryotic genomes. Bioinformatics 2007;23: 1061–1067. doi: 10.1093/bioinformatics/btm071 17332020 10.1093/bioinformatics/btm071

[64] Musacchia F, Basu S, Petrosino G, Salvemini M, Sanges R. Annocript: a flexible pipeline for the annotation of transcriptomes able to identify putative long noncoding RNAs. Bioinformatics. 2015;31: 2199–2201. doi: 10.1093/bioinformatics/btv106 25701574 10.1093/bioinformatics/btv106

[65] Marchler-Bauer A, Zheng C, Chitsaz F, Derbyshire MK, Geer LY, Geer RC, et al CDD: conserved domains and protein three-dimensional structure. Nucleic Acids Res. 2013;41: D348–352. doi: 10.1093/nar/gks1243 23197659 10.1093/nar/gks1243 PMC3531192

[66] Ashburner M, Ball CA, Blake JA, Botstein D, Butler H, Cherry JM, et al Gene ontology: tool for the unification of biology. The Gene Ontology Consortium. Nat Genet. 2000;25: 25–29. doi: 10.1038/75556 10802651 10.1038/75556 PMC3037419

[67] Bairocha A. The ENZYME database in 2000. Nucleic Acids Res. 2000;28: 304–305. 10592255 10.1093/nar/28.1.304 PMC102465

[68] Morgat A, Coissac E, Coudert E, Axelsen KB, Keller G, Bairoch A, et al UniPathway: a resource for the exploration and annotation of metabolic pathways. Nucleic Acids Res. 2012; 40 (Database issue): D761–769. doi: 10.1093/nar/gkr1023 22102589 10.1093/nar/gkr1023 PMC3245108

[69] Robinson MD, McCarthy DJ, Smyth GK. edgeR: a Bioconductor package for differential expression analysis of digital gene expression data. Bioinformatics 2010;26: 139–140. doi: 10.1093/bioinformatics/btp616 19910308 10.1093/bioinformatics/btp616 PMC2796818

[70] Chun JT, Gioio AE, Crispino M, Giuditta A, Kaplan BB. Characterization of squid enolase mRNA: sequence analysis, tissue distribution, and axonal localization. Neurochem Res. 1995;20:923–930. 8587650 10.1007/BF00970738

[71] Livak KJ, Schmittgen TD. Analysis of relative gene expression data using real-time quantitative PCR and the 2^-ΔΔ CT^ Method. Methods 2001;25:402–408. doi: 10.1006/meth.2001.1262 11846609 10.1006/meth.2001.1262

[72] Wernersson R. Virtual Ribosome—a comprehensive translation tool with support for sequence feature integration. Nucleic Acids Res. 2006;34: W385–W388. doi: 10.1093/nar/gkl252 16845033 10.1093/nar/gkl252 PMC1538826

[73] Saitou N, Nei M. The neighbor-joining method: a new method for reconstructing phylogenetic trees. Mol Biol Evol. 1987;4: 406–425. 3447015 10.1093/oxfordjournals.molbev.a040454

[74] Robinson O, Dylus D, Dessimoz C. Phylo.io: Interactive Viewing and Comparison of Large Phylogenetic Trees on the Web. Mol Biol Evol. 2016;33: 2163–2166. doi: 10.1093/molbev/msw080 27189561 10.1093/molbev/msw080 PMC4948708

[75] Suzek BE, Huang H, McGarvey P, Mazumder R, Wu CH. UniRef: comprehensive and non-redundant UniProt reference clusters. Bioinformatics. 2007;23: 1282–1288. doi: 10.1093/bioinformatics/btm098 17379688 10.1093/bioinformatics/btm098

[76] Georgiou I, Noutsopoulos D, Dimitriadou E, Markopoulos G, Apergi A, Lazaros L, et al Retrotransposon RNA expression and evidence for retrotransposition events in human oocytes. Hum Mol Genet. 2009;18: 1221–1228. doi: 10.1093/hmg/ddp022 19147684 10.1093/hmg/ddp022

[77] Krebs HA. Rate control of the tricarboxylic acid cycle. Adv Enzyme Regul. 1970;8: 335–353. 4920378 10.1016/0065-2571(70)90028-2

[78] Shiwa M, Murayama T, Ogawa Y. Molecular cloning and characterization of ryanodine receptor from unfertilized sea urchin eggs. Am J Physiol Regul Integr Comp Physiol. 2002;282: R727–737. doi: 10.1152/ajpregu.00519.2001 11832393 10.1152/ajpregu.00519.2001

[79] Brown NR, Noble ME, Endicott JA, Garman EF, Wakatsuki S, Mitchell E, et al The crystal structure of cyclin A. Structure. 1995;15: 1235–1247. 10.1016/s0969-2126(01)00259-3 8591034

[80] Kelley LA, Mezulis S, Yates CM, Wass MN, Sternberg MJ. The Phyre2 web portal for protein modeling, prediction and analysis. Nat Protoc. 2015;10: 845–858. doi: 10.1038/nprot.2015.053 25950237 10.1038/nprot.2015.053 PMC5298202

[81] Schmittgen TD, Livak KJ. Analyzing real-time PCR data by the comparative C_T_ method. Nat Protoc. 2008;3: 1101–1108. 18546601 10.1038/nprot.2008.73

[82] Standart N, Minshull J, Pines J, Hunt T. Cyclin synthesis, modification and destruction during meiotic maturation of the starfish oocyte. Dev Biol 1987;124: 248–258. 15669148 10.1016/0012-1606(87)90476-3

[83] Okano-Uchida T, Sekiai T, Lee K, Okumura E, Tachibana K, Kishimoto T. In vivo regulation of cyclin A/Cdc2 and cyclin B/Cdc2 through meiotic and early cleavage cycles in starfish. Dev Biol. 1998;197: 39–53. 9578617 10.1006/dbio.1998.8881

[84] Poccia D, Wolff R, Kragh S, Williamson P. RNA synthesis in male pronuclei of the sea urchin. Biochim Biophys Acta. 1985;824: 349–356. 2580559 10.1016/0167-4781(85)90042-9

[85] Hart MW, Byrne M, Smith MJ. Molecular Phylogenetic Analysis of Life-History Evolution in Asterinid Starfish. Evolution 1997;51: 1848–1861. doi: 10.1111/j.1558-5646.1997.tb05108.x 28565117 10.1111/j.1558-5646.1997.tb05108.x

[86] Knott KE, Wray GA. Controversy and consensus in Asteroid systematics: New insights to ordinal and familial Relationships. Amer. Zool. 2000;40: 382–392.

[87] Hebert PD, Ratnasingham S, deWaard JR. Barcoding animal life: cytochrome c oxidase subunit 1 divergences among closely related species. Proc Biol Sci. 2003;270 Suppl 1: S96–S99. 12952648 10.1098/rsbl.2003.0025 PMC1698023

[88] Foltz DW, Bolton MT, Kelley SP, Kelley BD, Nguyen AT. Combined mitochondrial and nuclear sequences support the monophyly of forcipulatacean sea stars. Mol Phylogenet Evol. 2007;43: 627–634. doi: 10.1016/j.ympev.2006.10.012 17113315 10.1016/j.ympev.2006.10.012

[89] Roux MM, Townley IK, Raisch M, Reade A, Bradham C, Humphreys G, et al A functional genomic and proteomic perspective of sea urchin calcium signaling and egg activation. Dev Biol. 2006;300: 416–433. doi: 10.1016/j.ydbio.2006.09.006 17054939 10.1016/j.ydbio.2006.09.006

[90] Parekh AB, Putney JW. Store-operated calcium channels. Physiol Rev. 2005;85: 757–810. doi: 10.1152/physrev.00057.2003 15788710 10.1152/physrev.00057.2003

[91] Morgan DO. Cyclin-dependent kinases: engines, clocks, and microprocessors. Annu Rev Cell Dev Biol. 1997;13:261–291. doi: 10.1146/annurev.cellbio.13.1.261 9442875 10.1146/annurev.cellbio.13.1.261

[92] Murray AW. Recycling the cell cycle: cyclins revisited. Cell. 2004;116: 221–234. 14744433 10.1016/s0092-8674(03)01080-8

[93] Malumbres M, Barbacid M. Mammalian cyclin-dependent kinases. Trends Biochem Sci. 2005;30: 630–641. doi: 10.1016/j.tibs.2005.09.005 16236519 10.1016/j.tibs.2005.09.005

[94] Meyerson M, Harlow E. Identification of G1 kinase activity for cdk6, a novel cyclin D partner. Mol Cell Biol. 1994;14: 2077–2086. 8114739 10.1128/mcb.14.3.2077-2086.1994 PMC358568

[95] Khleif SN, DeGregori J, Yee CL, Otterson GA, Kaye FJ, Nevins JR, et al Inhibition of cyclin D-CDK4/CDK6 activity is associated with an E2F-mediated induction of cyclin kinase inhibitor activity. Proc Natl Acad Sci U S A. 1996;93: 4350–4354. 8633069 10.1073/pnas.93.9.4350 PMC39540

[96] Moore JC, Sumerel JL, Schnackenberg BJ, Nichols JA, Wikramanayake A, Wessel GM, et al Cyclin D and cdk4 are required for normal development beyond the blastula stage in sea urchin embryos. Mol Cell Biol. 2002;22: 4863–4875. doi: 10.1128/MCB.22.13.4863-4875.2002 12052892 10.1128/MCB.22.13.4863-4875.2002 PMC133905

[97] Draetta G, Luca F, Westendorf J, Brizuela L, Ruderman J, Beach D. Cdc2 protein kinase is complexed with both cyclin A and B: evidence for proteolytic inactivation of MPF. Cell. 1989;56: 829–838. 2538242 10.1016/0092-8674(89)90687-9

[98] Galas S, Barakat H, Dorée M, Picard A. A nuclear factor required for specific translation of cyclin B may control the timing of first meiotic cleavage in starfish oocytes. Mol Biol Cell. 1993;4: 1295–1306. 7513215 10.1091/mbc.4.12.1295 PMC275765

[99] Martindale MQ, Brandhorst BP. Translational changes induced by 1-methyladenine in anucleate starfish oocytes. Dev Biol. 1984;101: 512–515. 6692993 10.1016/0012-1606(84)90164-7

[100] Rosenthal ET, Brandhorst BP, Ruderman JV. Translationally mediated changes in patterns of protein synthesis during maturation of starfish oocytes. Dev Biol. 1982;91: 215–220. 7201427 10.1016/0012-1606(82)90026-4

[101] Nakano E, Monroy A. Incorporation of S35-methionine in the cell fractions of sea urchin eggs and embryos. Exp Cell Res. 1958;14: 236–244. 13524222 10.1016/0014-4827(58)90182-4

[102] Epel D. Protein synthesis in sea urchin eggs: a "late" response to fertilization. PNAS 1967;57: 899–906. 5232369 10.1073/pnas.57.4.899 PMC224632

[103] Rosenthal ET, Hunt T, Ruderman JV. Selective translation of mRNA controls the pattern of protein synthesis during early development of the surf clam, *Spisula solidissima*. Cell 1980;20: 487–494. 7190072 10.1016/0092-8674(80)90635-2

[104] Goustin AS, Wilt FH. Protein synthesis, polyribosomes, and peptide elongation in early development of *Strongylocentrotus purpuratus*. Dev Biol 1981;82: 32–40. 7227637 10.1016/0012-1606(81)90426-7

[105] Frei RE, Schultz GA, Church RB. Qualitative and quantitative changes in protein synthesis occur at the 8-16-cell stage of embryogenesis in the cow. J Reprod Fertil. 1989;86: 637–641. 2760892 10.1530/jrf.0.0860637

[106] Gross KW, Jacobs-Lorena M, Baglioni C, Gross PR. Cell-free translation of maternal messenger RNA from sea urchin eggs. Proc Natl Acad Sci U S A. 1973;70: 2614–2618. 4582192 10.1073/pnas.70.9.2614 PMC427067

[107] Wilt FH. Polyadenylation of maternal RNA of sea urchin eggs after fertilization. Proc Natl Acad Sci U S A 1973;70: 2345–3249. 4525169 10.1073/pnas.70.8.2345 PMC433732

[108] Danilchik MV, Yablonka-Reuveni Z, Moon RT, Reed SK, Hille MB. Separate ribosomal pools in sea urchin embryos: ammonia activates a movement between pools. Biochemistry 1986;17: 3696–3702. 10.1021/bi00360a033 3718954

[109] Paris J, Richter JD. Maturation-specific polyadenylation and translational control: diversity of cytoplasmic polyadenylation elements, influence of poly(A) tail size, and formation of stable polyadenylation complexes. Mol Cell Biol. 1990;10: 5634–5645. 1700272 10.1128/mcb.10.11.5634-5645.1990 PMC361324

[110] Ochi H, Chiba K. Hormonal stimulation of starfish oocytes induces partial degradation of the 3' termini of cyclin B mRNAs with oligo(U) tails, followed by poly(A) elongation. RNA 2016;22: 822–829. doi: 10.1261/rna.054882.115 27048146 10.1261/rna.054882.115 PMC4878609

[111] Hennebert E, Leroy B, Wattiez R, Ladurner P. An integrated transcriptomic and proteomic analysis of sea star epidermal secretions identifies proteins involved in defense and adhesion. J Proteomics. 2015;128: 83–91. doi: 10.1016/j.jprot.2015.07.002 26171724 10.1016/j.jprot.2015.07.002

[112] Levner MH. RNA transcription in mature sea urchin eggs. Exp Cell Res. 1974;85: 296–302. 4827870 10.1016/0014-4827(74)90130-x

[113] Brandhorst BP. Simultaneous synthesis, translation, and storage of mRNA including histone mRNA in sea urchin eggs. Dev Biol. 1980;79: 139–148. 7190942 10.1016/0012-1606(80)90079-2

[114] Wang Z, Gerstein M, Snyder M. RNA-Seq: a revolutionary tool for transcriptomics. Nat Rev Genet. 2009;10: 57–63. doi: 10.1038/nrg2484 19015660 10.1038/nrg2484 PMC2949280

[115] Mestdagh P, Hartmann N, Baeriswyl L, Andreasen D, Bernard N, Chen C, et al Evaluation of quantitative miRNA expression platforms in the microRNA quality control (miRQC) study. Nat Methods. 2014;11: 809–815. doi: 10.1038/nmeth.3014 24973947 10.1038/nmeth.3014

[116] Watanabe N, Vande Woude GF, Ikawa Y, Sagata N. Specific proteolysis of the c-mos proto-oncogene product by calpain on fertilization of Xenopus eggs. Nature. 1989;342: 505–511. doi: 10.1038/342505a0 2555717 10.1038/342505a0

[117] Sagata N, Watanabe N, Vande Woude GF, Ikawa Y. The c-mos proto-oncogene product is a cytostatic factor responsible for meiotic arrest in vertebrate eggs. Nature. 1989;342: 512–518. doi: 10.1038/342512a0 2531292 10.1038/342512a0

[118] Gale AS. Phylogeny and classification of the Asteroidea (Echinodermata). Zool. Soc. Linnean Soc. 1987;89: 107–132.

[119] Moore WS. Inferring phylogenies from mtdna variation: mitochondrial-gene trees versus nuclear-gene trees. Evolution 1995;49: 718–726. doi: 10.1111/j.1558-5646.1995.tb02308.x 28565131 10.1111/j.1558-5646.1995.tb02308.x

[120] Lafay B, Smith AB, Christen R. A combined morphological and molecular approach to the phylogeny of asteroids (Asteroidea: Echinodermata). Syst. Biol. 1995;44: 190–208.

[121] Wada H, Komatsu M, Satoh N. Mitochondrial rDNA phylogeny of the asteroidea suggests the primitiveness of the paxillosida. Mol Phylogenet Evol. 1996;6: 97–106. doi: 10.1006/mpev.1996.0062 8812310 10.1006/mpev.1996.0062

[122] Matsubara M, Komatsu M, Araki T, Asakawa S, Yokobori S, Watanabe K, et al The phylogenetic status of Paxillosida (Asteroidea) based on complete mitochondrial DNA sequences. Mol Phylogenet Evol. 2005;36: 598–605. doi: 10.1016/j.ympev.2005.03.018 15878829 10.1016/j.ympev.2005.03.018

