# Supplementary figures and images for: De novo assembly of a transcriptome from the eggs and early embryos of Astropecten aranciacus
Source: PLoS One. 2017 Sep 5;12(9):e0184090. doi: 10.1371/journal.pone.0184090 (PMC5584759; doi:10.1371/journal.pone.0184090)

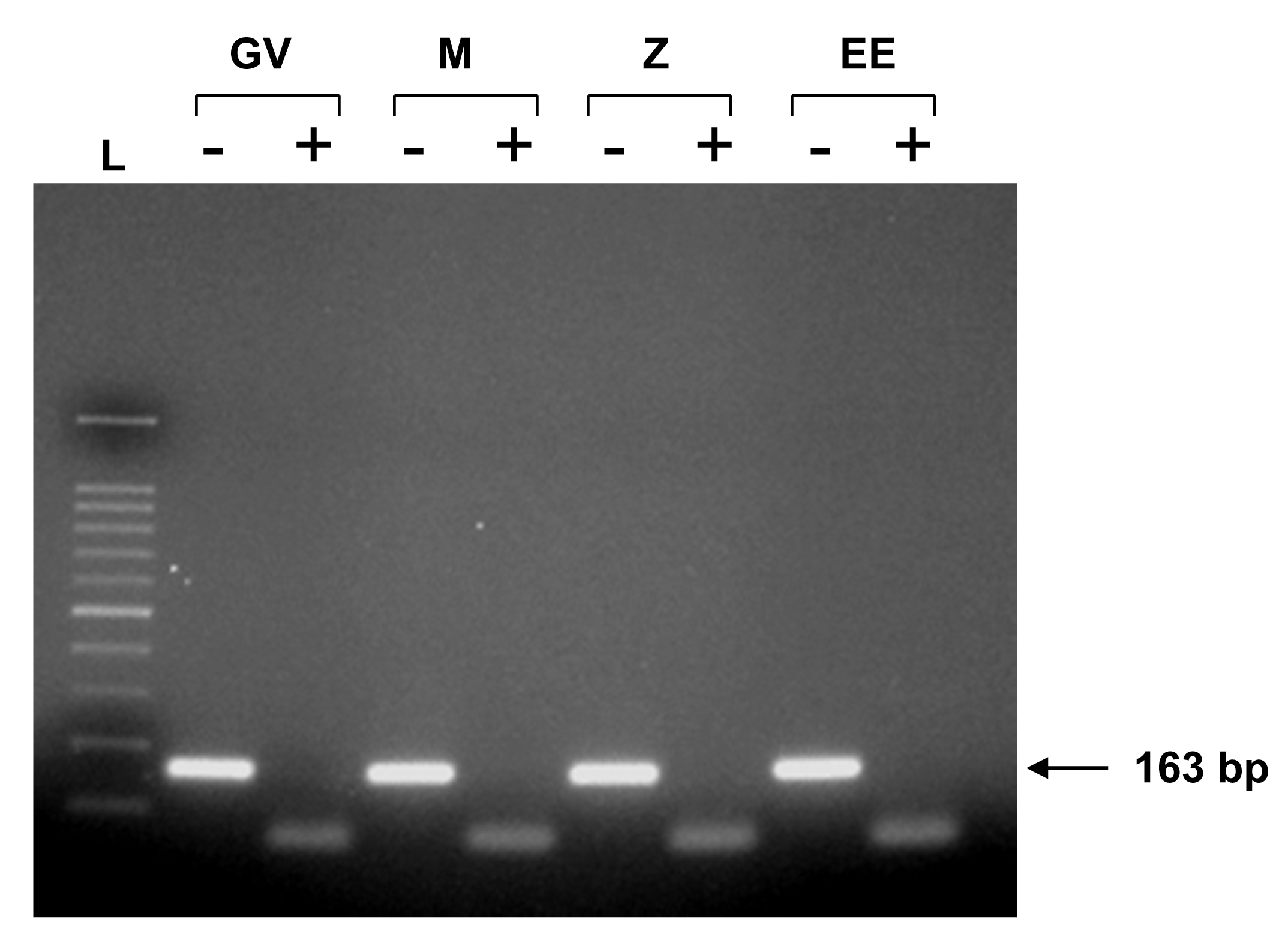

Supplement: S1 Fig — Biological replicates of RNA samples representing each stage, i.e. GV oocytes (GV), mature eggs (M), Zygotes (Z) and early embryos (EE), were pooled together and converted to cDNA. As a negative control, aliquots of RNA were pretreated with DNAse-free RNAse prior to cDNA synthesis, as described previously [70]. While cyclin A primers (S1 Table) produced abundant amplicons in RT-PCR after saturating number of cycles (= 40), no detectable amplicons were present in the PCR product from the samples pretreated with RNAse, suggesting that virtually all PCR products originated from RNA, not DNA. Lanes. L, 100 bp DNA ladder; (+) RNAse-pretreated samples; (-) Samples without RNA pretreatment. (TIF) [file pone.0184090.s002.tif]

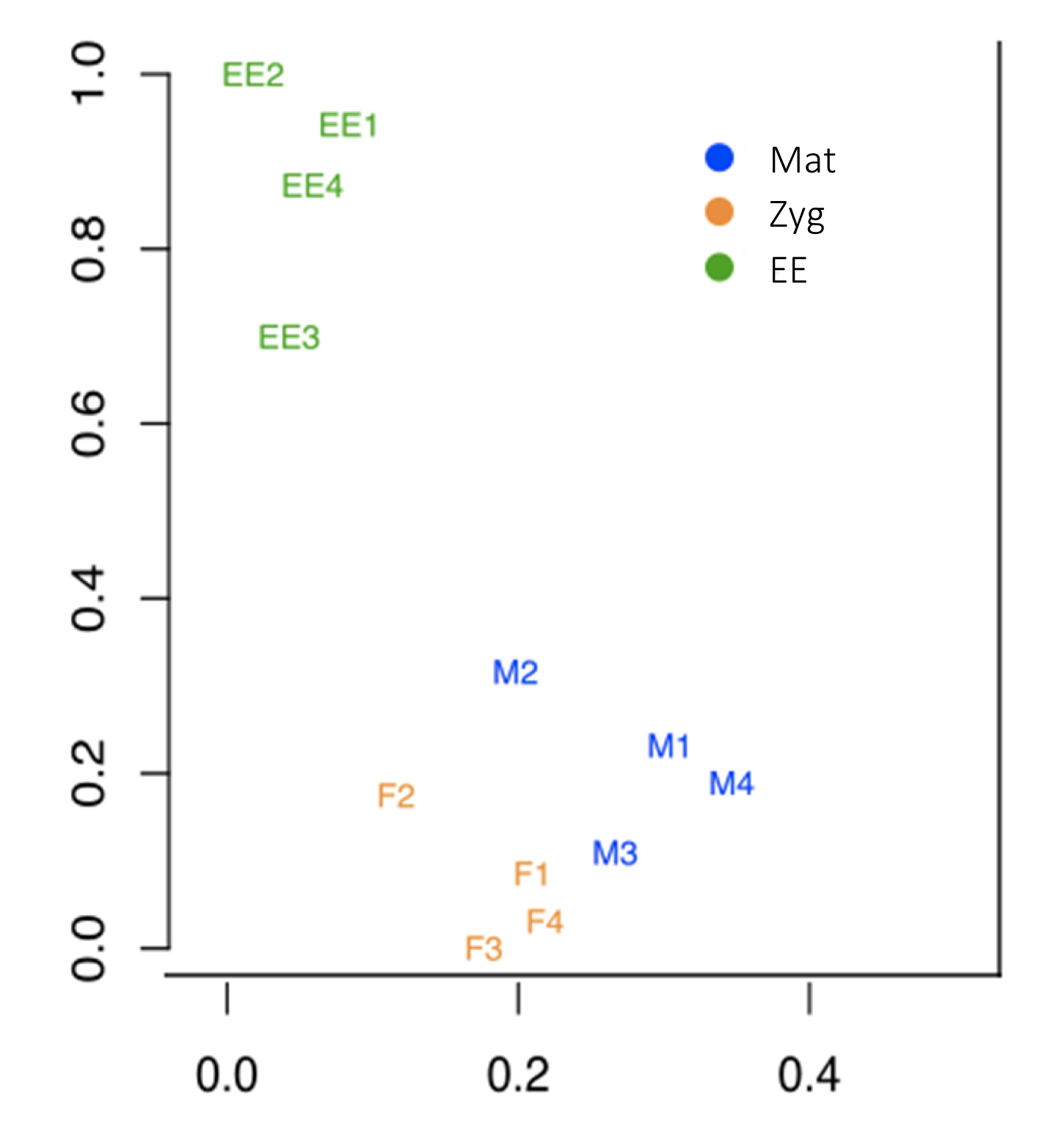

Supplement: S2 Fig — The FPKM (Fragments Per Kilobase of transcript per Million mapped reads) read counts were clustered using the cmdscale function in R. It is evident that the biological replicates of each stage were converged in a restricted area, but were appreciably separated from the converged replicates of other stages. Reflecting the little changes of gene expression between Mature eggs (Mat) and Zygotes (Zyg, fertilized eggs), the converged area defined by M1-M4 was relatively close to that of F1-F4. The data from the GV-stage were not included in this cluster analysis because they were processed as pooled samples. (TIF) [file pone.0184090.s003.tif]
